# Optogenetic stimulation of the primary visual cortex drives activity in the visual association cortex

**DOI:** 10.1016/j.crneur.2023.100087

**Published:** 2023-04-08

**Authors:** Michael Ortiz-Rios, Beshoy Agayby, Fabien Balezeau, Marcus Haag, Samy Rima, Jaime Cadena-Valencia, Michael C. Schmid

**Affiliations:** a Biosciences Institute, Henry Wellcome Building, Medical School, Framlington Place, Newcastle upon Tyne, NE2 4HH, UK; b Faculty of Science and Medicine, University of Fribourg, Chemin du Musée 5, 1700, Fribourg, Switzerland; c Functional Imaging Laboratory, Deutsches Primatenzentrum (DPZ), Leibniz-Institut für Primatenforschung, Göttingen, Germany

**Keywords:** Optogenetics, fMRI, Laminar electrophysiology, Macaque, Visual cortex, V1, Vision

## Abstract

Developing optogenetic methods for research in non-human primates (NHP) is important for translational neuroscience and for delineating brain function with unprecedented specificity. Here we assess, in macaque monkeys, the selectivity by which optogenetic stimulation of the primary visual cortex (V1) drives the local laminar and widespread cortical connectivity related to visual perception. Towards this end, we transfected neurons with light-sensitive channelrhodopsin in dorsal V1. fMRI revealed that optogenetic stimulation of V1 using blue light at 40 Hz increased functional activity in the visual association cortex, including areas V2/V3, V4, motion-sensitive area MT and frontal eye fields, although nonspecific heating and eye movement contributions to this effect could not be ruled out. Neurophysiology and immunohistochemistry analyses confirmed optogenetic modulation of spiking activity and opsin expression with the strongest expression in layer 4-B in V1. Stimulating this pathway during a perceptual decision task effectively elicited a phosphene percept in the receptive field of the stimulated neurons in one monkey. Taken together, our findings demonstrate the great potential of optogenetic methods to drive the large-scale cortical circuits of the primate brain with high functional and spatial specificity.

## Highlights

• Macaque V1 optogenetic stimulation drives visual association cortex.
• Stronger activation under stimulation at 40 Hz compared to lower frequencies.
• V1 optogenetic activation generates a phosphene, ie an artificial visual percept.
• The findings serve as a first step for optogenetics based visual cortical prosthesis.

## Graphical abstract

**Figure:**
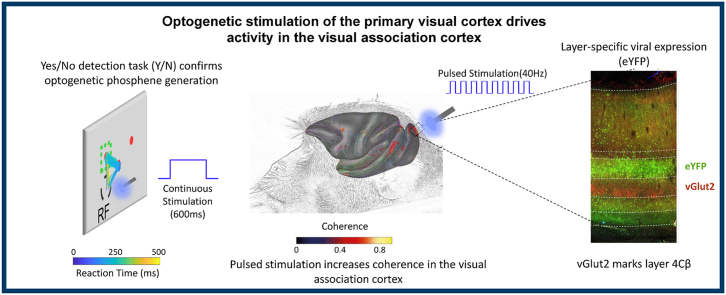

## 1 Introduction

Recent advances in optogenetic applications are unravelling functional circuit properties with greater neuronal specificity than traditional methods, such as electrical microstimulation and pharmacology, and therefore show great promise in contributing to a refined understanding of neural circuit function in the healthy as well as the diseased brain. However, translating the success of optogenetic methods from rodents to the primate brain has proven challenging. As a result, while electrical stimulation is routinely applied with great success in research and clinical settings to influence sensory-motor function and cognition, studies that use optogenetics successfully to manipulate behaviour in NHP remain limited (El-Shamayleh and Horwitz, 2019; Galvan et al., 2017; Tremblay et al., 2020). Delineating how optogenetic stimulation affects the functional circuitry of the primate brain, both at the local and global level, is therefore essential for achieving a deeper understanding of how optogenetic methods may successfully influence behaviour (Ju et al., 2018; Klink et al., 2021).

Here we focus on the macaque visual system, and specifically on the primary visual cortex (V1), which has been a long-standing target for delineating the neural basis of conscious vision (Leopold, 2012; Tong, 2003). Its high accessibility for implants combined with a retinotopic organisation that supports high-resolution vision are important reasons why V1 is regularly made the target for the development of prosthetic methods aimed at restoring visual function lost due to retinal disease (Beauchamp et al., 2020; Bosking et al., 2017; Tehovnik et al., 2009). V1 is the first cortical area known to integrate visual information within its laminar microcircuitry, before relaying visual information to higher-order cortical regions, broadly organised into dorsal ‘where’ and ventral ‘what’ pathways concerned with motion perception and object recognition respectively (Callaway, 1998; Casagrande et al., 1994; Felleman and Van Essen, 1991; Hubel and Wiesel, 1968; Ungerleider, 1982). While the anatomical connections of these cortical streams are well-established from tract tracing studies, it is not well understood how neuronal activity functionally propagates through the multiple hierarchical stages of the circuitry *in-vivo*. Previous interventional studies based on microstimulation of V1 were unable to dissociate the cortico-cortical activity, likely due to electrical current spread within V1 (Tolias et al., 2005a). Importantly, while some studies showed that microstimulation induces one synapse depolarization (Brock et al., 2013; Sultan et al., 2011; Logothetis et al., 2010a), others suggest that microstimulation-induced activity can propagate from V1 polysynaptically into multiple higher cortico-cortical regions (Fehérvári et al., 2015; Fehérvári and Yagi, 2016; Klink et al., 2017). Here, we predicted that cell-targeted optogenetic stimulation of V1 might reveal a more specific picture of cortico-cortical pathway connectivity. Specifically, from the laminar organisation of V1, we hypothesised that selective stimulation of the excitatory neurons of the granular layer 4-B, via optogenetics, could lead to an excitatory cortico-cortical output into projection zones of V2–V3 and MT along the cortical dorsal stream (Boyd and Casagrande, 1999; Elston and Rosa, 1997; Nassi and Callaway, 2007; Shipp and Zeki, 1989; Tigges et al., 1981; Weisenhorn et al., 1995). To test this hypothesis we combined layer targeted optogenetic stimulation (Klein et al., 2016) with the large-scale brain mapping technique functional magnetic resonance imaging (fMRI) to examine the distal as well as the local effects of stimulation (Ju et al., 2018; Gerits et al., 2012; Ohayon et al., 2013). In what follows, we report on the results from this investigation, showing BOLD fMRI activity in several areas of the visual association cortex following 40 Hz optogenetic stimulation of V1. We conclude with the presentation of preliminary results from one monkey that this circuit-specific activation approach was successful in eliciting a low contrast visual percept (‘phosphene’).

## 2 Materials and methods

### 2.1 Subjects

Four female rhesus monkeys (*Macaca mulatta*) were used to obtain opto-fMRI data (VL, 6 years old, weighing 7 kg; DP, 6 years old, weighing 9 kg; AL, 5 years old, weighing 9 kg; FL, 4 years old, weighing 6 kg). Surgical and anaesthesia procedures, postoperative care, and implant methods were described in detail in a previous manuscript (Ortiz-Rios et al., 2018, 2021). The UK Home Office approved all procedures, and procedures comply with the Animal Scientific Procedures Act (1986) on the care and use of animals in research and the European Directive on the protection of animals used in research (2010/63/EU).

### 2.2 Viral vectors and injections

The viral vector used, AAV9/5-hSyn-ChR2(H134R)-eYFP, was an adeno-associated virus (AAV), serotypes 5 and 9, carrying the neuron-specific human synapsin promoter (hSyn) and the humanised channelrhodopsin with the H134R mutation for optogenetic activation (hChR2(H134R)) alongside the enhanced yellow fluorescent protein (eYFP) for expression visualisation. Injections were made in the right hemisphere (see Supp. Fig. 1A for timeline).

**Fig. 1:**
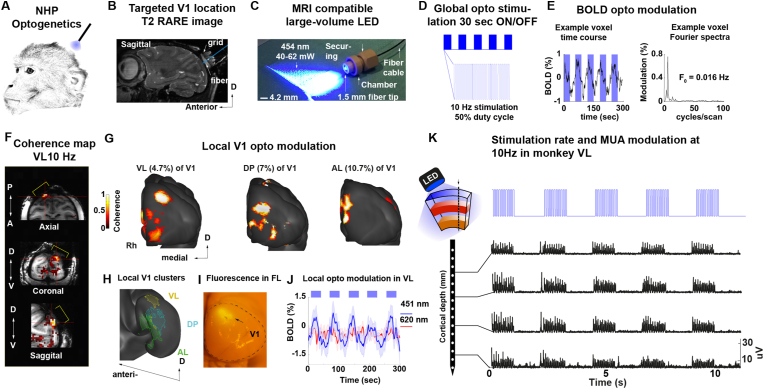
**Optogenetic stimulation drives the local BOLD modulation and spiking activity in transfected V1 neurons. A**. Optogenetic stimulation of V1 in awake NHPs. **B**. T2-RARE sagittal image showing the saline-filled chamber along with the centre coordinate of the optical fibre placement on monkey VL. **C**. MRI compatible LED fibre system with a tip diameter of 1.5 mm creating a 60° light beam that covered the full diameter (1.5 cm) of the craniotomy (at 40–62 mW power). **D**. Blue light stimulation (451 nm) at 10 Hz with a 50% duty cycle (50 ms pulse width) with 30-sec long stimulation on/off block pattern. **E**. BOLD signal modulation and power spectrum of an example voxel in V1 along with the voxel modulation peak at 0.016 Hz or 1 cycle/60 sec. **F**. In-volume activation map of monkey VL with the yellow brackets indicating the chamber region. **G**. Pial surface reconstruction of each individual monkey showing the model-free activation map based on the coherence between each voxel BOLD signal modulation and the stimulation paradigm (coherence threshold >0.3). The extent of V1 cortical activation (50–120 mm^2^) is summarised in Supplementary Table 1. **H**. Summary of the local response clustered area across three of the macaque monkeys (VL, DP and AL) shown on the inflated surface of the D99 macaque template. **I**. Post-mortem brain of monkey FL showing the native eyfp fluorescence after surface light stimulation with blue light on the opercular region of V1. **J**. Plot of the mean and ± std time course of each voxel as percent signal change obtained from the local clustered region of monkey VL for both blue (451 nm; blue trace) and red-light (620 nm; red trace). Note the wavelength dependence of the BOLD modulation **K**. Electrophysiological based optogenetic modulation of the transfected opercular region in V1 of monkey VL. Top left panel shows the schematic approach for recordings during opto stimulation. Multiunit activity during 15 s (on and off) optogenetic stimulation delivered at 10 Hz. Individual plots show the multiunit activity across channels located at different depths perpendicular to the cortical sheet. (For interpretation of the references to colour in this figure legend, the reader is referred to the Web version of this article.)

A surgical craniotomy was performed over dorsomedial V1 under general anaesthesia followed by an injection of an average volume of 24 μl of the viral solution (VL: 25.5 μl, DP: 25 μl, AL: 22 μl, FL: 24.5 μl of AAV9-hSyn-ChR2-eYFP (UPenn Lot: CS0964 based on Addgene 26973P, titre: 1.03e13 GC/ml). The construct was loaded into a 10 μl Nanofil syringe (World Precision Instruments) attached to a bevelled 34 GA needle (World Precision Instruments) prior to injections into the cortex under microscopic control using a microinjection system (UMP3-1, SYS-Micro 4; World Precision Instruments). To increase optogenetic responses after one year, animal DP was reinjected at ∼4 mm lateral and posterior of the first injection site using 28.5 μl of the same construct repackaged in serotype AAV5, which provided a higher titre (AAV5-hSyn-ChR2-eYFP, UPenn Lot: CS1078 based on Addgene 26973P, titre: 3.828e13 GC/ml). All injections were made in five different locations in a plus-shaped pattern within the chamber and separated by 2 mm horizontally and vertically (see example in Supp. Fig. 1B of monkey VL). On rare occasions, the injection site was slightly modified to avoid the penetration of blood vessels. To improve viral expression across different cortical laminae, injections were made at three different depths (approx. 1500 μm, followed by 1000 μm and 500 μm, see Supp. Fig. 1C) from the surface of the dura. The intended injection depth was confirmed upon retraction by visualising the exit of the injection needle from the cortex. In each depth, we injected a volume of approx. 1500 nl at a rate of 4 nl/s (approx. 6 min of total injection time). To compensate for the pressure induced by the injection needle, we allowed the tissue to settle for 1–2 min after moving to each depth and also after each injection. Following the injection procedure, the craniotomy was covered with a custom-fit MRI- compatible recording chamber (Ortiz-Rios et al., 2018, 2021).

### 2.3 Optical stimulation parameters

For the fMRI experiments, light from an LED system (blue at 451 nm and red 626 nm, LED, Prizmatix UHP System) was delivered during 30s-blocks followed by 30s-blocks of no stimulation. For the ON block, the light was delivered as a train of square pulses for the duration of the block; pulse trains were delivered at 5 Hz, 10 Hz, and 40 Hz at 50% duty cycles. The power of the 451 nm LED was between 40 and 62 mW and 50 mW for the 626 nm LED. Power was measured at the tip of the fibre that would go inside the chamber and placed on top of the tissue using a photodiode power sensor and a digital power metre (PM100D, Thorlabs). Power levels were similar whether an integrating sphere or a standard photodiode power sensor was used.

For the electrophysiology experiments, the same LED systems (451 nm with power 52–56 mW, 628 nm with power 50 mW) were used for epidural stimulation in monkey VL. Light was delivered continuously or pulsed (5 Hz, 10 Hz and 40 Hz at 50% duty cycle) for the stimulation period (1s) with a randomised intertrial interval (ITI) (1–2s) with no stimulation. In monkey FL, blue light (LuxX 473 nm diode laser, Omicron Lighthub-4, power at 37.5–55 mW) was delivered intracortically via an embedded optical fibre in the electrode array instead of epidural stimulation. Power was measured at the tip of the fibre before being coupled to the probe. All power levels were measured and calibrated prior to each experiment. For continuous stimulation conditions, stimulation duration was limited to 300 ms to avoid any potential damage. For pulsed stimulation, stimulation parameters were the same as previously described. Red light (594 nm DPSS laser, Omicron Lighthub-4, power at ∼40 mW) was only tested continuously with a stimulation period of 300 ms. Triggers (TTL signal) to the Laser and LED systems were sent and recorded using a behavioural experiment software (MWorks) via an interface card (National Instruments).

### 2.4 MRI data acquisition

All MRI data was acquired from awake animals. During the scanning period for optical stimulation, animals remained calm in darkness. Animal behaviour was monitored using a camera. However this camera did not allow us to record the precise eye-movement patterns. As a result, it is possible that differences in eye movement patterns, elicited by different stimulation conditions, might have contributed to the results. A vertical 4.7 T magnet, running ParaVision 5.1 (Bruker, BioSpin GmbH, Ettlingen, Germany) and equipped with a 4-channel phase-array coil that covered the whole head (https://www.wkscientific.com), was used to acquire MR images. Imaging data consisted of two types of datasets: T1 for anatomical analyses and echo-planar imaging (EPI) for functional analyses (Fig. 1).

For the first experiment in monkey VL.LV1 and one session in FL (FL.Ia1) we used a magnetization-prepared Rapid gradient echo (MP-RAGE) sequence to acquire anatomical (T1) images: FA (°) = 30; TE (ms) = 3.95; TR (ms) = 2000; TI (ms) = 750; Accel. Factor = 1; RIM-RO (px) = 176; RIM-PH (px) = 176; RIM-SL (px) = 72; RR-RO (mm) = 0.6; RR-PH (mm) = 0.6; RR-SL (mm) = 0.6; TA (h:m:s) = 0:14:48. For functional data on VL.LV1 and FL.Ia1 sessions we used a gradient-echo (GE) EPI sequence was used to acquire BOLD signal modulation for functional mapping with the following parameters: FA (°) = 65; TE (ms) = 21; TR (ms) = 1500; BW (Hz/Px) = 1704; ES (ms) = 58; Accel. Factor = 2; RR-RO (mm) = 1.2; RR-PH (mm) = 1.2; RR-SL (mm) = 1.2; RIM-RO (px) = 88; RIM-PH (px) = 88; RIM-SL (px) = 31; Nacq = 200; TA (h:m:s) = 0:5:00.

For all subsequent sessions we increased the resolution to obtain more detailed maps of the local activation region. For all monkeys and sessions (VL.MQ1, VL.M31, DP.NJ1, DP.Qq1, AL.SD1, AL.SI1, FL.Uv1, FL.Uy1) we used the following anatomical and functional parameters: MP-RAGE sequence for anatomical images: FA (°) = 30; TE (ms) = 5.42; TR (ms) = 2000; TI (ms) = 750; Accel. Factor = 1; RIM-RO (px) = 70; RIM-PH (px) = 82; RIM-SL (px) = 26; RR-RO (mm) = 0.27; RR-PH (mm) = 0.25; RR-SL (mm) = 1.4; TA (h:m:s) = 0:20:48. For functional data we used a gradient-echo (GE) EPI sequence with the following parameters: FA (°) = 65; TE (ms) = 21; TR (ms) = 1700; BW (Hz/Px) = 3409; ES (ms) = 58; Accel. Factor = 2.3; RR-RO (mm) = 0.79; RR-PH (mm) = 0.79; RR-SL (mm) = 1.4; RIM-RO (px) = 70; RIM-PH (px) = 70; RIM-SL (px) = 20; Nacq = 176; TA (h:m:s) = 0:5:00.

For chamber visualisation (Fig. 1B), we used a Rapid Imaging With refocused Echoes (RARE) sequence with the following parameters: TEeff (ms) = 57; TR (ms) = 12500; FA (°) = 90/180; BW (Hz/px) = 284; RR-RO (mm) = 0.61; RR-PH (mm) = 0.61; RR-SL (mm) = 0.62; RIM-RO (px) = 176; RIM-PH (px) = 176; RIM-SL (px) = 92; Nacq = 5; RARE Factor = 8; TA (h:m:s) = 0:22:55.

### 2.5 fMRI data analyses

All pre-processing analyses were conducted using open software provided in the packages of AFNI (Cox, 1996), SUMA (Saad et al., 2004). For anatomical scans, the D99 atlas (Reveley et al., 2017a) was used to re-create an in-session anatomical surrogate brain of each monkey. White matter and grey matter were obtained from the in-session atlas ROIs parcellation of the warped D99 template. The resulting masks were then used to render a white, pial, and flattened surface using Freesurfer (Dale et al. Sereno). For displaying the ROIs onto the surface, we first created a colour mappable niml file of the atlas volume using the AFNI program (3dVol2Surf). In SUMA, we then displayed the contours of each ROI on the surface along with the activation maps. Pre-processing of fMRI time series followed standard preprocessing steps which included: slice-timing correction, motion correction, spatial-smoothing (3dmerge, 1.5 mm fwhm), and mean scaling of the time series. 3dDeconvolve was used for linear least-squares detrending to remove nonspecific variations (i.e., scanner drift) and regression that included the overall white matter resulting in a residual time-series which were then used for coherence analyses.

Coherence analyses were then performed on the residual time series by measuring the ratio between the amplitude at the fundamental frequency to the signal variance, ranging between 0 and 1 (Brewer et al., 2002). The measure of coherence is

$${C=A\left(f_{0}\right)∕\left(\sum_{f_{0}+\Delta f∕2}^{f_{0}+\Delta f∕2}{A\left(f\right)}^{2}\right)}^{1∕2}$$

where, f₀ is the stimulus frequency, $A\left(f_{₀}\right)$ the amplitude of the signal at that frequency, A(f) the amplitude of the harmonic term at the voxel temporal frequency f and ᅀf the bandwidth of frequencies in cycles/scan around the fundamental frequency f₀. For optogenetic stimulation conditions, f₀ corresponds to one cycle (1/60 s = 0.016 Hz) and ᅀf corresponds to the frequencies around the fundamental frequency (see Fig. 1D and E).

### 2.6 Electrophysiological data acquisition and analysis

Laminar probes were used to record neural activity across the cortical layers in two animals (VL & FL). In monkey VL, dual-shaft laminar Atlas probes (Atlas Neuro, Leuven, Belgium) with 16 electrodes in each shaft (150 μm electrode spacing, 50 μm electrode diameter) were used. An optic fibre (1.5 mm diameter, 0.5 N.A.) was placed outside the cortex to deliver light from an external LED similar to the fMRI experiment (451 nm for stimulation, 626 nm for control). In monkey FL, laminar Plexon S-probes (Plexon Inc., Texas, USA) with 24 contacts (100 μm electrode spacing, 15 μm electrode diameter) were used. For intracortical stimulation, an embedded optical fibre (46 μm inner diameter) in the upper third of the probe, between channels 8 and 9, was used for light delivery from the external laser system described earlier.

Raw electrophysiological signals were recorded using a Blackock recording system (BlackRock Microsystems, Inc.) and sampled at 30 kS/s. Data analyses were done in Matlab using the Fieldtrip toolbox (Oostenveld et al., 2011) and custom scripts. The CSDplotter toolbox (Pettersen et al., 2006) was used to calculate the current source density (CSD) profiles of the visually evoked responses using the standard CSD method. LFP signals were obtained by downsampling the raw signal to 500 Hz and then applying a notch filter at 50 Hz to remove any line noise. Multiunit activity (MUA) signals were obtained by applying a high pass filter at 250 Hz to the raw signal and then units were extracted using a threshold that is 2.5–3.5 std of the channel average. The envelope of multiunit activity (MUAe) was obtained by applying a high pass filter at 150 Hz, rectifying and then downsampling the signal to 500 Hz (Supèr and Roelfsema, 2004). Z-scores of neural signals were calculated based on the mean and standard deviation (std) of the baseline period before stimulation onset. Optogenetic stimulation trials were extracted based on the onset of the TTL trigger signal.

For epidural stimulation using blue light, light onset artefacts were present and were removed manually during MUA extraction, however it was not possible in MUAe. For intracortical stimulation, no light artefacts were observed in the MUA/MUAe signals so MUAe was used in analyses. Response onsets for visual and optogenetically modulated responses were calculated by detecting when the signal crosses a threshold defined as: baseline average + 4*std.

### 2.7 Visual stimulation and behavioural task

Animals were positioned at a distance of 84 cm in front of a ViewPIXX computer screen (RR: 120 Hz, VPIXX technologies), allowing for the presentation of stimuli at ±16° horizontally and ±9.5° vertically. Behavioural tasks were programmed and executed using MWorks (MWorks) with custom scripts that used the MWorks Experiment Language (MWEL).

At the start of each experimental session, an animal's eye movements were calibrated to the eye tracking system using a visual calibration task in which the animal performed saccades to and fixated (500ms) on visual stimuli that appeared at pseudo-random locations (12 points) on the screen. The saccade response windows were iteratively reduced until a precession adequate precision (<2° visual angle) was achieved. Upon successful trials, the animals received a reward in the form of fruit juice or water.

Automatic receptive field (RF) mapping was performed in order to identify the spatial location of the minimum RF of underlying neuronal populations. In the task, animals were required to maintain central fixation (1° window for 1s). Meanwhile, black squares (0.5/1° size) were presented (>10 times, 100ms each) on a 5 × 5 grid in the lower left quadrant of the visual field while electrophysiological activity was recorded. The RF centre was estimated based on the evoked MUAe at each grid location.

A yes/no task (Y/N) was used to test for optogenetically generated phosphenes. The Y/N task was used such that the animal reports the existence or absence of stimuli by making a saccade to one of two targets. In the task, central fixation (within a window <1°, for 500–1250 ms) was followed by the appearance of two saccade targets (0.3° red discs) in the upper left and upper right part of the screen (∼4° from the centre). The saccade targets were accompanied by one of the following equally probable conditions (El-Shamayleh and Horwitz, 2019): the presentation of a 1° white disc with varying contrasts on the left side of the central fixation point inside the RF that was previously determined (visual trial) (Galvan et al., 2017), no visual or optical stimuli (control condition, catch trial) or (Tremblay et al., 2020) continuous optogenetic stimulation (opto trial). The visual and optogenetic stimuli were extinguished immediately when the animal made a saccade to either target or after 600ms in the absence of any response with the trial considered as ignored and discarded. The animal was initially trained on a version of the task in which 50% visual and 50% catch trials were randomly interleaved. In visual trials, the animals received a reward for making a saccade to the left target indicating stimulus detection trial while, in catch trials, the animal received a reward for making a saccade to the right target indicating the reporting of stimuli absence. Later on, following optogenetic transfection, sessions consisted of an equal 33% of catch, visual and optogenetic stimulation trials. In opto trials, animals received a reward if they performed a saccade to the left target similar to visual trials. d’ was used as a measure of sensitivity. d’ is the difference between the z-transforms of hits and false alarms. A value of d’ was calculated for the opto and visual trials. Catch trials were used to estimate false alarms and correct rejections.

### 2.8 Eye movement analyses for the behavioural task

Eye position was recorded with Eyelink 1000 plus with a sampling rate of 1000 Hz; the raw signal was calibrated and converted to visual degrees by Mworks based on the calibration task. We detected eye movements automatically with a usually employed velocity-based algorithm (see (Engbert and Kliegl, 2003), for details). Velocity was calculated using a 5ms moving window according to the equation:

$${\vec{v}}_{n}=\frac{{\vec{x}}_{n+2}+{\vec{x}}_{n+1}-{\vec{x}}_{n-1}-{\vec{x}}_{x-2}}{6\Delta t}$$

Where ${\vec{x}}_{n}$ denotes an eye location sample and Δt denotes the time between samples of 1 ms. An eye movement was classified as a saccade if velocity increased beyond 6 times the median estimator ($\sigma_{x,y}$) with the detection threshold ($\eta_{x,y}$) according to the equation:

$$\eta_{x,y}=\lambda \sigma_{x,y}$$

with λ of 6. Reaction times (RTs) were calculated as the time interval between the onset of the stimulus and the onset of the saccade (first saccade in case of multiple saccades). No latencies were below 100 ms.

### 2.9 Histological analysis of brain slices

Once sufficient experimental data was gathered, animals were anaesthetised with an overdose of anaesthetic and then perfused through the heart with phosphate-buffered saline (37°, pH 7.4), followed by paraformaldehyde (40%, pH 7.4). The brains were then carefully removed and placed in Paraformaldehyde (40%, pH 7.4) over night. Subsequently, the brain was placed in increasing sucrose concentrations (10%, 20%, 30%) for cryoprotection.

Tissue was then cut on a freezing microtome (50 μm sections) and then stained using Cresyl violet before being mounted on microscopy slides. A subset of sections was also stained using a standard immunohistological protocol. Sections were incubated in a trisodium citrate buffer (pH 6, 0.1 M, 1% horse serum, 1% goat serum, 0.3% TritonX) for 60 min. Sections were then incubated with a primary antibody for the vesicular glutamate transporter (vGlut2, MAB5504, Merckmillipore) used to label cells in the cortical layer V1 4C beta (Brodmann's nomenclature), as has been shown previously (Balaram and Kaas, 2014), diluted in PBS for 24 h, before being treated with AlexaFluor conjugated secondary antibodies for 2 h. Sections were then mounted on frosted microscope slides, covered with a mounting medium conjugated with a nuclear cell body marker (Fluoroshield TM with DAPI, Merck) and protected using standard coverglass. Following treatment, representative sections were imaged using a fluorescence microscope (DM6B, Leica Navigator).

## 3 Results

### 3.1 Optogenetic V1 stimulation induces local BOLD and spiking activity

Our first aim was to map the local neural activation induced by the optogenetic stimulation of V1. Viral injections (∼24 μl of *AVV9/5-hSyn-hChR2*) were made at multiple depths and sites of dorsal opercular V1 (approx. around 5–7° of visual eccentricity, lower left visual hemifield representation). The injection resulted in an estimated virally transfected area of approx. 12 mm^2^ (see Table 1 and Supp. Fig. 1B). For stimulation, a large-volume LED illuminator was placed epidurally inside an MRI-compatible recording chamber (Ortiz-Rios et al., 2018) implanted over the occipital lobe for chronic access to V1 (Fig. 1A and B). The illuminator consisted of a 1.5 mm diameter optical fibre with an effective 60° light beam (Fig. 1C) that was connected to an external LED producing blue light (451 nm) for effective stimulation of channelrhodopsin (ChR2).

**Table 1:** Summary of fMRI activation.

	Blue light		Red light		Movie	
*Monkey*	Activation cluster mean coherence	Activation cluster sigma coherence	Activation cluster mean coherence	Activation cluster sigma coherence	Activation cluster mean coherence	Activation cluster sigma coherence
*VL*	0.4	0.2	0.14	0.08	0.62	0.19
*DP*	0.55	0.28	–	–	0.33	0.18
*AL*	0.45	0.16	0.21	0.09	0.31	0.09

	**Blue light**		**Red light**		**Movie**	
***Monkey***	**Activation cluster mean phase**	**Activation cluster sigma phase**	**Activation cluster mean phase**	**Activation cluster sigma phase**	**Activation cluster mean phase**	**Activation cluster sigma phase**
*VL*	196.78	30.62	151.53	44.2	172.33	35.62
*DP*	150.22	23.15	–	–	147.95	24.46
*AL*	103.23	26.81	131.2	35.37	214.91	39.83

***Monkey***	**Total number active voxels**	**Total number of V1 voxels**	**V1 volume (mm3)**	**Percent activation of V1 volume**	**Activation volume (mm3)**	**voxel size mm3**
*VL*	1840	72199	1949.37	2.55%	49.68	0.3
*DP*	2816	71383	1927.34	3.94%	76.03	0.3
*AL*	3709	83422	2252.4	4.45%	100.43	0.3

***Monkey***	**Total number active surface voxels**	**Total number of surface V1 voxels**	**V1 area (mm2)**	**Percent activation of V1 area**	**Activation area (mm2)**	**voxel size mm2**
*AL*	535	11493	1034.27	4.65%	48.15	0.3 × 0.3
*DP*	808	11401	1026.09	7.08%	72.72	0.3 × 0.3
*AL*	1354	12585	1132.65	10.75%	121.86	0.3 × 0.3

*Monkey*	Activation cluster mean %CNR	Activation cluster sigma %CNR	Activation cluster mean %CNR	Activation cluster sigma %CNR	Activation cluster mean %CNR	Activation cluster sigma %CNR
*VL*	0.7	0.1	0.4	0.2	2.0	0.12
*DP*	0.76	0.14	–	–	1.6	0.4
*AL*	0.94	0.2	0.05	0.1	–	–

The effectiveness of the elicited optogenetic stimulation was then first assessed during opto-fMRI experiments using the vertical MRI scanner acquiring images at high-resolution (1.2 mm isotropic voxel size) while monkeys were at rest and allowed to freely move their eyes. Blue light stimulation parameters consisted of 451 nm wavelength set at 10 Hz with 50% duty cycle and a power range of 40–62 mW. Examination of the BOLD signal time course in a voxel located at the V1 chamber position in response to pulsed blue light (Fig. 1D) revealed its systematic modulation during periods of optogenetic stimulation (see left panel on Fig. 1E). In order to quantify the BOLD modulation in response to optogenetic stimulation, we used voxel-based coherence analysis as the analytical method of choice, since the hemodynamic response function (HRF) of opto-fMRI in macaques has not been formally characterised. To this end, the strength of the BOLD response was assessed by first calculating the frequency-resolved power spectrum and then determining the coherence between the BOLD peak frequency response (cycles/volume) and the stimulus repetition rate (0.016 Hz = 1/60 s) (see right panel in Fig. 1E).

From the opto-fMRI experiments, we observed significant BOLD modulation (cluster size >10 voxels, coherence >0.35, n runs = 4) in response to blue-light stimulation of the opercular area of V1 (Fig. 1F, for threshold coherence map overlaid on T1 anatomy). Comparison with visual stimulation (Supp. Fig. 2A) revealed that opercular V1 was also active during natural vision and that visual stimulation resulted in stronger BOLD modulation compared to the one from optogenetic stimulation under these experimental conditions. To better visualise the activation pattern elicited in V1 by optogenetic stimulation, we overlaid the BOLD activation map on a 3D surface reconstruction of the occipital lobe (Fig. 1G). Across the three imaged monkeys, the local activation mapping revealed an activation focus over the dorsal opercular V1, with a size of 50–120 mm^2^ corresponding to about 5–10% of the V1 surface area (see Table 1). Interestingly, in all three monkeys, weaker activation could also be observed in the more ventral part of V1, possibly reflecting the local dorso-ventral connectivity (Casagrande et al., 1994). The spatial proximity of the activated V1 zones across the three monkeys (VL, DP and AL) is shown in the standardised D99 macaque brain template (Reveley et al., 2017b) in Fig. 1H and compared to the *ex-vivo* fluorescence pattern from an additional monkey (FL) in which a similar injection approach was performed (Fig. 1I). Thus, these initial results demonstrated the effectiveness of the optogenetic approach in driving the local V1 BOLD response using fMRI. Our next aim was then to establish the extent to which the observed BOLD response pattern was wavelength-specific and whether optogenetic stimulation could also drive local spiking activity.

**Fig. 2:**
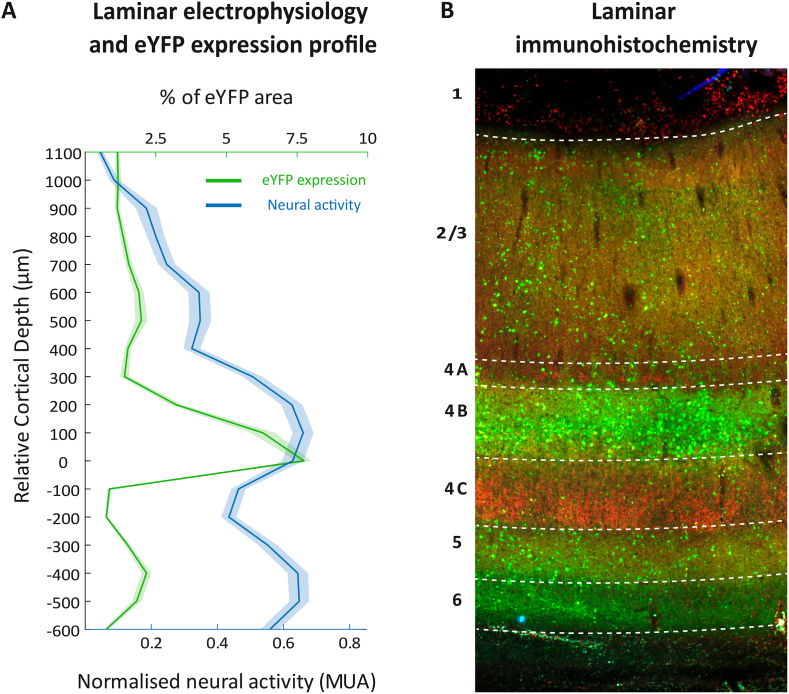
**V1 neural activity closely matches immunohistochemistry expression profile across cortical layers in monkey FL**. **A**. Sustained (100–300ms) laminar activation pattern (blue) for continuous (300 ms) optogenetic stimulation and area percentage expressing eYFP (green) as a function of relative cortical depth (aligned to layer 4C). The laminar activation pattern was calculated for each session and then normalised by the maximum firing rate for that session. Alignment was calculated based on the earliest response to a visual stimulus. **B**. Laminar profile of V1 near the injection site. eYFP expression of the optogenetic construct can be seen in green, layer 4C (particularly 4C beta) was co-stained using an anti-vGlut2 antibody using standard immunohistochemistry (red). (For interpretation of the references to colour in this figure legend, the reader is referred to the Web version of this article.)

### 3.2 V1 BOLD and neural responses to 451 nm but not to 626 nm stimulation

An important confound to consider in optogenetics is the potential for heat build-up in the brain tissue due to light absorption (Stujenske et al., 2015). In particular with opto-fMRI, heat-induced negative BOLD signal modulations have been observed in rodent animal models (Christie et al., 2013; Desai et al., 2010). While we observed only positive BOLD signal modulation during optogenetic stimulation, we attempted to address the potential effects of heat by carrying out additional control experiments.

We aimed to determine if the local BOLD response was specific to blue-light stimulation (451 nm), or if the response could also be evoked by red light (626 nm) stimulation. In independent ex-vivo control experiments, we had determined that both wavelengths exerted similar temperature effects under our testing conditions (Supp. Fig 3). We assumed that the BOLD responses should only be driven by blue-light to which ChR2 is sensitive (Boyden et al., 2005). In contrast, stimulation with red light should resulted in no response given that ChR2 is not sensitive to 626 nm wavelength light stimulation. To address the potential confound of wavelength-specific effects, we carried out experiments in the same ChR2-transfected animals. During experiments we alternated between blue and red light stimulation runs keeping the stimulation power constant at 50 mW and pulsed at 10 Hz, 50% duty cycle for both wavelengths. In monkey VL (session M31), we found again a local positive BOLD response in V1 to blue light (451 nm, 50 mW at 10 Hz; cluster size >10 voxels, coherence >0.35, n runs = 4; Fig. 1J, blue line; see coherence map without threshold in Supp. Fig. 4A and C). During the same session, but on alternating runs, we also stimulated with red-light. Here, we found no activation (626 nm, 50 mW at 10 Hz; cluster size >10 voxels, coherence >0.35, n runs = 4; Fig. 1J, red line; see coherence map without threshold in Supp. Fig. 4B and C) at the same local V1 site previously activated by blue-light. On average across experimental runs, the optogenetic target region in V1 displayed lower contrast-to-noise ratios (CNR) for red-light (mean CNR %, 0.4 ± std 0.2) as compared to blue light (mean CNR %, 0.7 ± std 0.2). Similarly, blue light stimulation resulted in a significant coherence activation cluster (n runs = 6, coherence of 0.4, ± std 0.2), while red light resulted in non-significant modulation (n runs = 6, coherence of 0.14, ± std 0.08). To assess the reliability of this effect, we carried out additional opto-fMRI experiments in monkey AL and found significant local BOLD modulation (see map without threshold and with threshold in Supp. Fig. 4D; cluster size >10 voxels, coherence >0.35, n runs = 4) only for blue-light (451 nm, 50 mW at 10 Hz) but not for red-light (626 nm, 50 mW at 10 Hz). Similarly, only blue-light stimulation resulted in significant BOLD modulation (n runs = 4, mean coherence 0.45, ± std 0.21, mean CNR % 0.94 ± std 0.2) while red-light resulted in no response modulation (n runs = 4, mean coherence 0.21, ± std 0.09, mean CNR % 0.05 ± std 0.1).

**Fig. 3:**
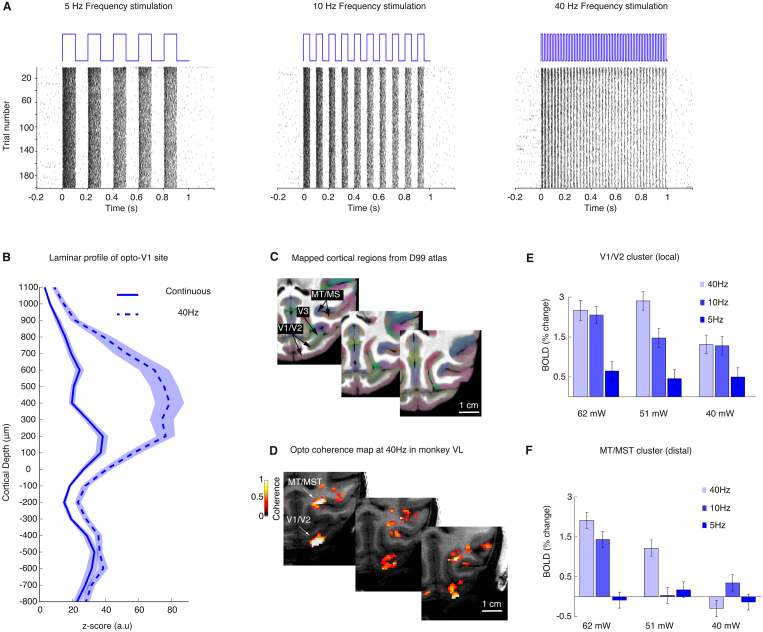
**Increases in extrastriate BOLD activity after increased frequency stimulation of opercular V1**. **A**. Example raster plots of neural activation from monkey FL shows reliable temporal modulation of spiking activity from optogenetic stimulation rate (5 Hz, left panel; 10 Hz, middle panel, and 40 Hz right panel). **B**. Laminar profile of the local V1 neural activation during continuous (solid line) and 40 Hz stimulation (dashed line) in monkey FL. The z-score reflects the neural activation strength as a function of cortical depth centred at the granular level (layer IV or 0 distance in mm). The pattern shows increased spiking modulation (mean z-score ± std) in supragranular layers of the transfected region in V1 as compared to continuous stimulation (blue line). **C**. Cortical regions form the D99 atlas (top panel) mapped onto the warped template of monkey VL. **D**. Maps of monkey VL showing significantly active regions which are labelled as follows: visual areas V1, V2 and V3 and motion-sensitive regions MT/MST. **E**. Average percent BOLD signal change of the V1/V2 local cluster region of monkey VL tested at three different power levels (40 mW, 51 mW and 62 mW) and three different frequency levels (5 Hz, 10 Hz, and 40 Hz). For similar data of frequency dependent fMRI modulation from monkeys DP and AL, see Supp. Fig. 8. B. **D**. Average percent signal change of the MT/MST distal cluster region as similarly shown in **D**. Note the strongest modulation occurs at maximum power levels and at 40 Hz frequency. (For interpretation of the references to colour in this figure legend, the reader is referred to the Web version of this article.)

**Fig. 4:**
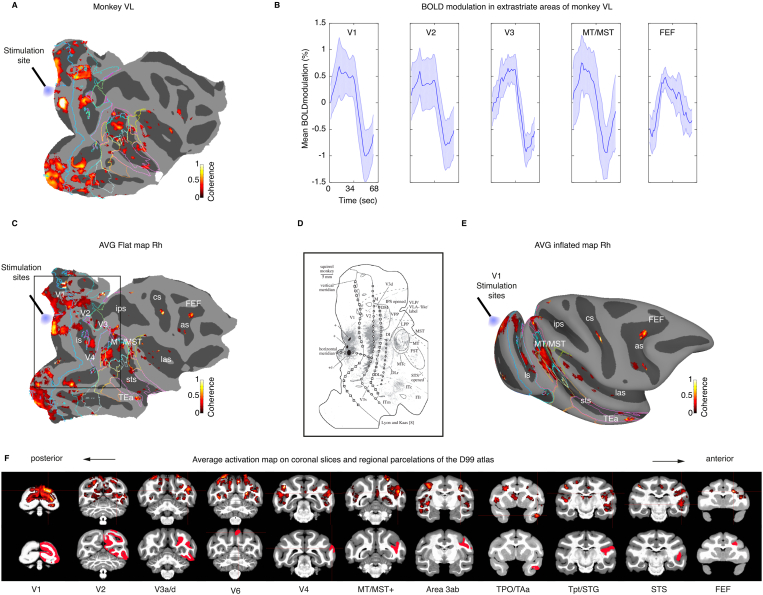
**V1 optogenetic stimulation drives BOLD activation in higher cortical areas**. **A**. Cortical activation map of monkey VL in response to optogenetic stimulation of opercular V1. **B**. Average single-cycle BOLD modulation across activated cortical regions showing extrastriate BOLD activity in V1, V2, V3 and motion complex regions MT/MST in example monkey VL with additional activity seen in area FEF. For additional maps in monkeys DP and AL see Supp. Fig. 11. **C** shows a flat map for the averaged activation map across monkeys VL, DP and AL (coherence >0.3, mean 0.46 > 0.2 ± std). The map shows cortical regions driven by optogenetic stimulation of opercular V1. LED fibre points to the stimulation sites in the opercular region, with a size of 50–120 mm^2^ corresponding to about 5–10% of V1. Activation outside V1 includes motion-sensitive regions MT/MST and area FEF in the frontal lobe. Sulci are displayed in dark grey while gyri are displayed in light grey. Contours show main visual and higher visual regions obtained from the D99 atlas. **D**. V1 tract-tracing map (Lyon and Kaas, 2002) showing a terminal labelling pattern in new world monkeys that is very similar to the activation map obtained with our opto-fMRI in macaques (panel C). **E**. Same map as in C, but on a 3D inflated surface. **F**. Same activation map as in C and E showing posterior to anterior clusters region in coronal slices along with the corresponding areal boundary map of the D99 atlas.

To further investigate the neurophysiological basis of the observed optogenetically induced BOLD fMRI response in V1, we used multi-contact electrode arrays in V1 to record the spiking activity of cortical neurons during optical stimulation of the transfected brain tissue. Optical stimulation was delivered epidurally as similarly performed during fMRI experiments (Fig. 1K). To represent neuronal spiking activity, we extracted one multiunit for each channel. In monkey VL, ten sites were sampled throughout five experimental sessions. In total, 104 units were extracted and out of which 85 units showed a significant increase in firing rate in response to a 1-s continuous pulse stimulation with blue light (451 nm, p < 0.05, Wilcoxon signed-rank test). The units extracted covered the whole depth of the cortex showing reliable neural activation across the V1 target zone. The response latencies of the modulated MUA were short (<10 ms, example Supp. Fig. 6) indicating a direct activation by blue light, a characteristic of ChR2 (Boyden et al., 2005).

In summary, with our initial experiments, we were able to determine that the local BOLD response underlying the injection sites reflected changes in neural activity specific to blue light and that heat-related effects were rather unlikely. Somewhat surprisingly, however, our initial fMRI assessment showed very little remote activation beyond V1. As frequency-dependent effects have been reported in studies using electrical stimulation in combination with neuroimaging (es-fMRI) (Kampe et al., 2000; Logothetis et al., 2010b; Murris et al., 2020; Van Camp et al., 2006), we reasoned that optogenetic stimulation frequency could be a mediating factor in driving activation into higher-level regions.

### 3.3 V1 neural activity closely matches layer specific immunohistological expression

To further examine the effects of optogenetic modulation on the V1 cortical microcircuit, we used laminar probes to examine the optogenetically modulated activity across the different layers of V1 of monkey FL. The laminar coverage of the probes was determined based on the CSD profiles of LFP and the MUA across channels in response to a visual stimulus. From these measures we identified the geniculate recipient layer, layer 4C, as the electrode contacts that carried the earliest current sink (Supp. Fig. 5A) and shortest MUA response latency (<50 ms, Supp. Fig. 5B) (Maier et al., 2010; Schroeder et al., 1998).

**Fig. 5:**
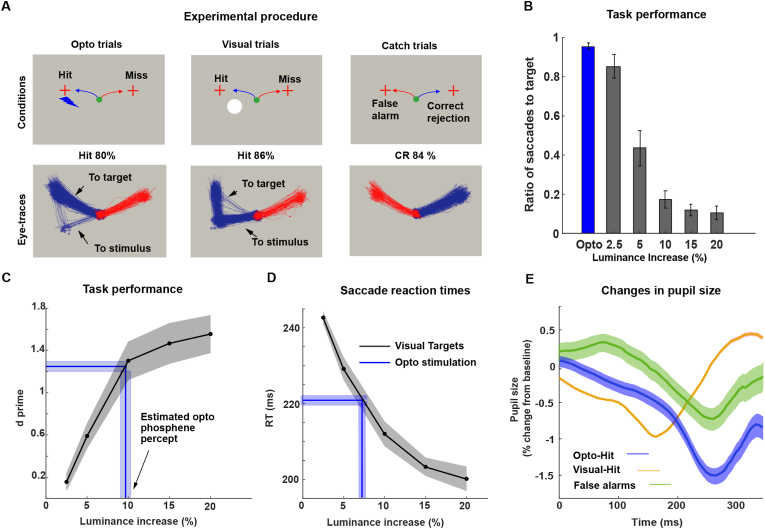
**Induction of a visual phosphene from optogenetic stimulation of primate V1 for monkey FL**. **A**. Schematic representation of the 3 experimental conditions (top panel) and associated eye movement trajectories in response to each stimulation condition (bottom panel). Note that optogenetic stimulation typically resulted in direct saccades to the response target, whereas saccadic reactions to visual stimulation were typically first directed at the visual stimulus, before continuing to the response target. **B** and **C**. Comparison of visual sensitivity **B** and saccadic reaction times **C** between the visual and optogenetic stimulation conditions. The blue line indicates the mean and SEM visual sensitivity to optogenetic stimulation. Projection of optogenetic to visual sensitivity and reaction time curves indicates that the optogenetically elicited phosphene likely corresponds to the percept evoked by a 5–10% luminance (increase over background) visual stimulus. **D**. Proportion of saccades to target vs to stimulus locations across the different experimental conditions. The majority of eye movements in response to optogenetic stimulation were directed to the target. The number of saccades to target decreased with increasing luminance levels in the visual condition. **E**. Comparison pupil size changes following visual (orange line) vs optogenetic (blue line) stimulation and catch trials (green line). Shading indicates standard error of means. (For interpretation of the references to colour in this figure legend, the reader is referred to the Web version of this article.)

For optogenetic stimulation, the laminar electrode was equipped with an embedded optical fibre allowing intracortical light delivery at 350–650 μm, as estimated from the relative cortical depth in monkey FL. Across 19 sessions, 354 out of 380 multi-units showed significant increase in firing rates (p < 0.05, Wilcoxon signed rank) in response to a continuous 300 ms stimulation pulse with blue light (473 nm) delivered via laser (see Methods). As expected, red-light (594 nm) did not affect neural activity (Supp. Fig. 6). Across experimental sessions, we sampled from locations up to 2 mm around the injection site. The averaged laminar profile of the sustained (100–300ms) MUAe response showed significant increases in firing rates across sessions (19/20 contacts p < 0.001, and one contact p = 0.05, Wilcoxon signed rank, right tailed test) with activation peaks in putative layers 4 B followed by layers 5 and 6 (Fig. 2A, blue line). Histological analyses of the V1 transfected region (Supp. Fig. 1E) of the same monkey confirmed an increase in percentage of eYFP positive cell expression in layers 4 B, 5 and 6 with sparser expression in layers 2/3 (Fig. 2B) resulting in a significant correlation between the electrophysiological response and histological expression profiles (Fig. 2A, Spearman's ⍴ = 0.5273, p = 0.026). eYFP fluorescence was also apparent in the V1 of the three other monkeys participating in the study (Supp. Fig. 7); however, the quality of the histological data was insufficient for a more detailed laminar analysis from these monkeys.

Taken together, our results with fMRI, electrophysiology and histology demonstrate a robust activation of the local V1 microcircuit, including, at least in one monkey, the strongest response modulation in layer 4 B that connects V1 to extrastriate cortical areas (Casagrande et al., 1994). Somewhat surprisingly, however, our initial fMRI assessment showed very little remote activation beyond V1. As frequency dependent effects have been reported in studies using electrical stimulation in combination with neuroimaging (es-fMRI (Kampe et al., 2000; Logothetis et al., 2010; Murris et al., 2020; Van Camp et al., 2006), we reasoned that optogenetic stimulation frequency could be a mediating factor in driving activation into higher-level regions.

### 3.4 Increasing V1 stimulation frequency drives extrastriate BOLD activity

We tested the effect of applying different optogenetic stimulation frequencies (5 Hz, 10 Hz, 40 Hz, 50% duty cycles) suited to activate ChR2 (Boyden et al., 2005) on the V1 laminar electrophysiological response level in monkey FL, in addition to determining frequency-dependent BOLD response modulation in monkeys VL, AL and DP. As predicted, we found that neural activity followed the optogenetic stimulation frequency closely (Fig. 3A). To evaluate the response pattern further, we calculated an averaged laminar activation profile for each frequency as previously done for continuous stimulation. For both 5 Hz and 10 Hz, the activation increases were small and uniform, and the laminar activation pattern was not different from continuous stimulation (Supp. Fig. 8A). However, stimulation delivered at 40 Hz caused an overall increase in activation and especially in the more superficial layers, which was not observed with continuous or lower frequency stimulation (Fig. 3B, Supp. Fig. 8A). As superficial layers are known to send their projections to higher order areas of visual association cortex (Casagrande et al., 1994), we took advantage of fMRI to map whether 40 Hz stimulation was more efficient in driving BOLD activation remotely. For these opto-fMRI measurements, the global stimulation design was kept constant at 30 s (ON and OFF), while the pulse rate was set at 50% duty cycle for all the frequencies (5 Hz, 10 Hz, and 40 Hz) and different power levels (40 mW, 51 mW and 62 mW) were tested. To identify active regions driven by the optogenetic stimulation we overlaid the D99 atlas (Reveley et al., 2017b) onto the warped template of our in-session anatomical scan (Fig. 3C). Focusing on the early striate and extrastriate cortex, we observed activation in areas V1/V2 and MT/MST under 40 Hz stimulation (Fig. 3D). A closer examination of the response magnitude revealed effects for both the stimulation amplitude as well as the frequency in monkey VL (Fig. 3 E, F). A similar frequency-dependent increase in fMRI activity was also observed in monkeys DP and AL (Supp. Fig. 8B). Importantly, at the strongest stimulation power of 62 mW and 40 Hz stimulation frequency, the BOLD response effect was more efficient than at lower stimulation frequencies. Independent control measurements of light intensity at the fibre output connected to the stimulation LED, confirmed the equivalence of power output for 10 and 40 Hz (Supp. Fig. 9) and a temperature increase for 5 and 40 Hz (Supp. Fig. 3). Our finding of increased opto-fMRI activation with higher stimulation frequencies is consistent with previous studies using electrical microstimulation in combination with fMRI (Kampe et al., 2000; Logothetis et al., 2010b; Murris et al., 2020; Van Camp et al., 2006).

### 3.5 Predominant dorsal stream BOLD activity after optogenetic stimulation of V1

Having established the effectiveness of 40 Hz stimulation for driving local and remote activation, we wondered in which areas of visual association cortex activation could be measured with fMRI and how robust such activation would be in our different monkeys. Fig. 4A shows the overall BOLD activation pattern in monkey VL in a cortical flat map for better visualisation. Optogenetic stimulation was effective in driving activation in several cortical areas of the visual system, including V1, V2, V3, MT/MST, V6 and FEF (Fig. 4B and F). Only very sparse activity was observed in the most anterior part of area V4 and more anteriorly along ventral stream regions of the superior temporal sulcus (STS). Additional activation could also be observed subcortically in the lateral geniculate nucleus (LGN) in monkey DP (Supp. Fig. 10). At the cortical level in V1, we observed some variation across animals in the overall cluster extent (50–120 mm^2^). This could be due to several factors, such as the transfection spread of AVV9 across the cortical sheet and or the polysynaptic propagation of functional activity. Despite slight variations with respect to the specific dorsal part of V1 being optogenetically transfected and stimulated, qualitatively very similar results with activation of extrastriate cortex were observed across the three monkeys (see Supp. Fig. 11 for flat maps on individual monkeys). This similarity in the cortical activation pattern is highlighted in the averaged activation map across the three investigated monkeys with predominant, though not exclusive, activation of areas belonging to the cortical dorsal stream (Fig. 4C and E and Supp. Fig. 11). Interestingly, despite the previously noted activation in ventral V1/V2, only limited activation could be observed in ventral stream regions beyond V1/V2 and the temporal lobe. These results from opto-fMRI appear qualitatively very similar to previous tracer-based characterization of V1 connectivity (Fig. 4D) (Lyon and Kaas, 2002). As this optogenetically induced response map with predominant dorsal stream activation only partially reflects the rich cortical activation pattern normally seen during free-viewing conditions (Ortiz-Rios et al., 2021; Selvanayagam et al., 2019; Russ and Leopold, 2015) (see Supp. Fig. 2A), we wondered whether the recruited network of brain areas might be sufficient to induce an artificial visual percept.

### 3.6 V1 optogenetic stimulation induces a visual “phosphene”

Monkey FL was successfully trained to perform the Y/N task (Fig. 5A, see Methods) design to test whether optogenetic stimulation could elicit a phosphene. The use of saccadic response targets in the upper portion of the screen was motivated by the need to disassociate the reporting from the stimulus position or the stimulated RF. Initially, the monkey was trained on a version of the task with only visual (50%) and catch (50%) trials. Once the monkey reliably reported its visual percept with a hit rate of >80% for both high-contrast visual stimulation (and above chance in the lower-contrast ones) and catch trial conditions, optogenetic stimulation of V1 was introduced with each trial type having equal probability (∼ = 33%). A critical component of the experimental procedure was to first electrophysiologically map the RF of optogenetically targeted neurons, before proceeding with the behavioural testing at this opto-RF location. The monkey apparently learned to generalise reporting from visual towards optogenetic trials on the first day of optogenetic testing: the monkey's overall performance during optogenetic stimulation was at 86% and therefore as high as during visual stimulation and catch trials (Fig. 5A, Supp. Fig. 13A). Comparing the performance during the opto trials with those during different visual luminance conditions demonstrated that the monkey had, on average across experiments, a sensitivity (d’, see Methods) of about 1.3 for optogenetic stimulation, matching the sensitivity to a <10% visual stimulus luminance increase (Fig. 5B). Similarly, reaction times of saccades (Fig. 5C) after visual stimulus onset decreased with increasing luminance and the reaction times of saccades after optogenetic stimulation were similar to low contrast visual stimulation. Thus, the monkey's report from optogenetic stimulation closely resembled his reports when a low contrast visual stimulus was present.

In order to establish whether a similar effect was also observable under implicit, physiological measures, we examined closely the monkey's eye movement response pattern (Fig. 5A and D). The testing regimen allowed the monkey to either make a saccade to the stimulus first and then to the response target, or straight to the target for both visual and opto conditions. In the visual condition, the macaque's saccade trajectory differed according to the luminance of the visual stimulus; under low contrast conditions, the monkey typically looked directly at the response target, whereas under high contrast conditions, the monkey first looked at the visual stimulus before making an additional saccade to the response target. In the opto condition, the majority of the monkey's saccades were aimed directly towards the response target (very similar to the visual 2.5% contrast condition), highlighting again the close similarity between performance during opto and low luminance contrast visual trials. In comparison to the trials with either opto or visual stimulation, the monkey made a saccade to the left ‘yes’ target in about 20% of catch trials (Supp. Fig 13B), and during these trials almost never looked at the RF location, but instead executed a direct saccade to the response target (Supp. Fig 13C). In addition to these observations on eye movements, we compared the effect of visual and opto stimulation on pupillary responses. Aligning the pupillary time-series with the onset of the stimulus indicates a pupillary constriction in response to optogenetic stimulation (Fig. 5E) that occurred with a ∼100 ms longer latency compared to visually induced pupillary constriction. To better compare the pupil response of opto vs visual stimulation, we aligned it with the onset of the saccade (Supp. Fig 13D). Consistent with performance and reaction time measures, also pupillary constriction varied with visual contrast level and the amount of constriction during optogenetic stimulation was very similar to the ones during stimulation at low visual contrast.

Taken together, the data from monkey FL demonstrate that V1 optogenetic stimulation can induce an artificial visual percept that appears to resemble the percept of a low-contrast stimulus.

## 4 Discussion

Despite the increasing importance of establishing optogenetic methods for investigations in non-human primates, the ability to initiate behaviour and cause changes in perception in this species remains a challenge. By performing a multi-modal assessment of our V1 optogenetic stimulation approach in macaque monkeys, we confirm the effectiveness and specificity of optogenetic stimulation for driving the V1 laminar circuitry and higher-order visual cortical areas. However, a limitation of our opto-fMRI approach is that non-specific heating and eye-movement contributions to the obtained BOLD activation maps can not entirely be ruled out. Preliminary results from one monkey indicate that this circuit-specific optogenetic activation proved suitable to elicit artificial visual percepts (‘phosphenes’). In our discussion, we highlight the benefits and limitations of using optogenetics for mapping the V1 microcircuitry and the large-scale cortical networks, before considering how this method can be optimally used to induce meaningful behaviour. We compare our findings with existing studies in which macaque V1 has been stimulated either with optogenetics or with electrical microstimulation and discuss how confounding effects, such as heating, might have contributed to our findings.

### 4.1 Specificity of activation maps with opto-vs es-fMRI

An important study by Tolias et al. reported for the first time the successful implementation of electrical microstimulation in combination with fMRI (‘es-fMRI’) as a method to map effective brain connectivity (Tolias et al., 2005b). From stimulating V1, the authors reported BOLD activation in the extrastriate cortex which closely resembles our findings with optogenetic stimulation (Fig. 4), including activation of V2/V3 and MT/MST. In our data, this V2/V3 and MT/MST activation pattern observed with fMRI is well predicted from the laminar electrophysiology and histological fluorescence profile with a peak in layer 4 B that we observed at least in one monkey (Fig. 2). As neurons in layer 4 B are known to project to areas V2 and MT (Boyd and Casagrande, 1999; Elston and Rosa, 1997; Nassi and Callaway, 2007; Shipp and Zeki, 1989; Tigges et al., 1981; Weisenhorn et al., 1995), the fMRI activation that we found in these extrastriate areas corresponds well to the observed V1 expression pattern. As V1 projects to V2 and MT both directly and indirectly (Boyd and Casagrande, 1999; Elston and Rosa, 1997; Nassi and Callaway, 2007; Shipp and Zeki, 1989; Tigges et al., 1981; Weisenhorn et al., 1995), both es- and opto-fMRI maps closely reflect this known connectivity with dorsal stream areas. Qualitatively quite similar to the results with es-fMRI, we also observed only very sparse fMRI activation of ventral stream regions such as V4 and more anterior regions of the STS concerned with object and face perception. On one hand this effect was expected from the laminar expression pattern peaking in layer 4 B. On the other hand, we found that 40 Hz stimulation appeared to overcome V1 laminar specificity and drive also layers projecting to ventral stream areas. At this point, we can only speculate about a greater contribution of neurons that are more sensitive to higher frequency stimulation. These neurons might be more abundant in the dorsal stream owing to the need to process movement at higher temporal resolution.

In addition to this predominantly parieto-occipital activation pattern, we also observed activation in the frontal eye field (FEF) in the prefrontal cortex. We attribute the lack of FEF activation in the Tolias study to the selection of RF coils or to the use of anaesthesia, which often tends to suppress activation especially in high level association cortex. The activation seen with opto-fMRI may arise polysynaptically via dorsal-stream activation, but could also arise from the direct V1 projection to FEF (Perkel et al., 1986). These methodological uncertainties notwithstanding, there is a large overlap between opto- and es-fMRI mapping results.

### 4.2 Opto-fMRI: effects of heating and stimulation frequency

A limitation of our fMRI results is that we can not entirely rule out heating effect contributions. From its initial report, opto-fMRI has been subjected to confounding heating effects (Stujenske et al., 2015; Christie et al., 2013; Lee et al., 2010; Desai et al., 2011; Arias-Gil et al., 2016; Schmid et al., 2017; Owen et al., 2019). Briefly, light delivery to the brain has been demonstrated to directly shift the frequency of the NMR signal and associated T1 and T2* images (Christie et al., 2013). Physiologically, heat-induced activations are typically reported as a decrease of the BOLD signal at the fibre tip and as reduced firing rates of neurons (Desai et al., 2011; Schmid et al., 2017; Owen et al., 2019). This is however not what we observed in our study; the BOLD signal activations we measured were positive and in the accompanying electrophysiology, we typically observed increases in multi-unit activity with short latencies. An ex-vivo test of temperature modulations of our epidural LED approach, used for the fMRI experiments, revealed, albeit clear modulation of temperature, that this was a rather limited effect and equal for blue (451 nm) and red (626 nm) light under these test conditions (Supp. Fig. 11). This is in contrast with the marked difference between blue and red light-induced activation effects that we observed with fMRI. While our comparison of the effects of blue vs red light stimulation on the BOLD response and electrophysiological signals thus makes heating effects appear unlikely, we can not entirely exclude such heating contributions to our fMRI results. fMRI measurements under blue light stimulation in a non-transfected cortex would have been a better control for heating (Stujenske et al., 2015). At the same time, applying pulsed stimulation as used in our study, is known to prevent heat build-up (Stujenske et al., 2015); this has been confirmed by an additional experiment in ex-vivo tissue (Supp. Fig. 3). Additionally, the increased response cannot be explained by a difference in the total power delivered at 40 Hz compared to the slower frequencies. While some light pulses exhibited an initial transient power increase, the duration and the number of such transients were minor (Supp. Fig. 9) and did not result in a significant difference in the total power delivered to the tissue. Here, it is also important to bear in mind that our study is only the second to report successful use of opto-fMRI in NHP, which contrasts with the routine use of optogenetics in non-primate species. The study by Gerits et al. successfully used optogenetic stimulation in FEF to map the large-scale effective connectivity from area FEF and successfully influenced saccadic eye movements (Gerits et al., 2012). In contrast, a study with a seemingly identical approach for FEF stimulation, found no optogenetically induced fMRI activation, though it was possible to drive neuronal responses and influence saccadic behaviour. Interestingly, Gerits et al. used 40 Hz optogenetic stimulation, whereas Ohayon et al. applied brief continuous stimulation pulses for optogenetic stimulation. Our findings here indicate that pulsed 40 Hz stimulation is more effective in driving electrophysiological activity in V1 superficial layers and for propagating activation to remote brain areas (Fig. 3). The benefit of high-frequency stimulation is well known from electrical stimulation studies (Logothetis et al., 2010a; Kampe et al., 2000; Murris et al., 2020; Van Camp et al., 2006) and might help to explain the difference in mapping effective connectivity across NHP opto-fMRI studies. While the temporal resolution of ChR2 is limited, future studies should consider testing functional connectivity using opsins with faster kinetics, such as Chronos or Chrimson (Klapoetke et al., 2014) enable higher-frequency stimulation or switch to step-function opsins requiring only transient stimulation.

### 4.3 Using optogenetics in V1 to induce a visual phosphene

In stark contrast to the many studies employing electrical stimulation techniques with great success, only very limited evidence exists in NHPs that demonstrates the capacity to affect behaviour from optogenetic stimulation of the cortex. At the level of V1, the pioneering study by Jazaheri et al. using ChR2 demonstrated how spontaneous eye movements tended to be directed to the receptive field of optogenetically identified neurons (Jazayeri et al., 2012). While this finding corroborated earlier observations obtained with electrical stimulation (Tehovnik et al., 2003), it remained unclear what the monkey perceived from such stimulation. While a recent study did not report whether eye movements or a percept could be elicited from optogenetic stimulation of V1, the authors demonstrated how the stimulation could improve the detectability of weak visual stimuli, provided there was a match in the stimulus properties and the tuning for this property of the optogenetically targeted neurons (Andrei et al., 2019). However, Andrei et al. used a different virus and promoter, which could explain the difference from our findings. In addition, the laminar stimulation approach might have contributed to our success in eliciting a visual phosphene. Additionally, a study by De et al. recently showed how optogenetic V1 inactivation disrupted eye movements and reduced stimulus visibility by more than 50% during a perceptual choice task (De et al., 2020). Finally, a recent study by Chen and colleagues found that optogenetic stimulation of V1 in the presence of a visual stimulus resulted in masking effects similar to those observed between multiple visual stimuli (Chen et al., 2022). This provided evidence that V1 optogenetic stimulation indeed influences visual perception. However, the authors do not report optogenetically generated phosphenes and the animals were trained to ignore anything but visual stimuli. Therefore, it is not clear whether optogenetic stimulation on its own would have elicited a phosphene in that study. Here, our results in one monkey (Fig. 5) fill this important gap in the literature; stimulating V1 optogenetically induced a visual percept (‘phosphene’) which the monkey reliably reported with a hit rate of over 80%. Interestingly, in addition to its effect on explicit perceptual reports, optogenetic stimulation also induced pupillary constriction. For visual trials, pupil constriction increased with the increased luminance which could be attributed to the pupillary light reflex (PLR) (Clarke et al., 2003); however, we do not believe that the change in pupil size induced by optogenetic stimulation is directly activating the PLR circuitry. PLR responses are generally locked to the onset of the luminance increase (May and Warren, 2020; Pong and Fuchs, 2000), however we found the change in pupil diameter to be time-locked to the saccade onset. It has been previously shown that the pupil also reacts to imagined stimuli (Sulutvedt et al., 2018; Laeng and Sulutvedt, 2014). As such, we interpret the change of pupil size following optogenetic stimulation as a reflection of increased localised saliency as pupil constriction increases with stimuli saliency even for auditory stimuli (Corneil and Munoz, 2014; Wilschut and Mathôt, 2022). A recent study found similar patterns of increasing pupil constriction accompanied by decreasing RTs (Pandey and Ray, 2021) suggesting that those two metrics are related and reflect the saliency of a stimulus.

Gaining access to such implicit performance measurements might help in future studies aimed at identifying behavioural consequences from optogenetic stimulation. In an effort to better understand the perceptual quality of the evoked phosphene, we related the monkey's performance during optogenetic stimulation to his response pattern under visual stimulation at varied luminance contrast levels. The performance measurements along with the eye movement patterns indicate that the monkey most likely perceived a phosphene that resembles a low-contrast visual stimulus. The sensitivity measure d’ as well as reaction times are comparable to those of a 10% luminance contrast visual stimulus. The saccade patterns are comparable to even lower contrasts; however, it is not possible to make a saccade to the exact phosphene location since it will move with the shift in gaze while the visual stimulus remains static on the screen, which at least in part might explain the higher percentage of direct saccades to targets for opto trials. This finding is very similar to observations made with electrical stimulation under comparable test conditions (Schiller et al., 2011). In that study by Schiller et al. a two-alternative forced-choice (2AFC) revealed that the electrically generated phosphene is also similar to a visual stimulus with a low contrast visual stimulus even at higher stimulation currents. Interestingly, it is also similar to the effects observed when V1 was optogenetically inhibited (Rajalingham et al., 2021); Rajalingham and colleagues found that when a visual stimulus is paired with optogenetic inhibition, the choice probability decreases to match that of a visual stimulus with a 10–15% decrease in visual signal. While the viral promoter and light delivery approach are different from our study, light was delivered continuously, similar to our paradigm. Naturally, the question then arises of how such a phosphene could be strengthened and how it could be turned into something more useful for visual function. Based on our assessment of stimulation frequency and amplitude effects on the recruitment of activation in higher-order association areas, as well as the local increased activation, we believe it might be preferable to apply 40 Hz stimulation also for behavioural testing. Since the weaker modulation in response to continuous optical stimulation was enough to reliably generate a visual percept, the much higher modulation resulting from higher frequency pulsed modulation might potentially induce a stronger phosphene.

In summary, our results offer a first insight into how the primary visual cortex and its projection to higher association areas can be driven with optogenetic stimulation in order to elicit an elementary visual percept. On a more general level, our findings also speak to the importance of delineating the complex primate cortical architecture with the high functional specificity that optogenetic methods offer.

## CRediT authorship contribution statement

**Michael Ortiz-Rios:** Conceptualization, Methodology, Formal analysis, Investigation, Writing – original draft. **Beshoy Agayby:** Methodology, Conceptualization, Formal analysis, Investigation, Software, Writing – original draft. **Michael C. Schmid:** Conceptualization, Funding acquisition, Project administration, Resources, Supervision, Writing – original draft.

## Declaration of competing interest

The authors declare that they have no known competing financial interests or personal relationships that could have appeared to influence the work reported in this paper.

## Data Availability

Data will be made available on request.
