# Supplementary material for: Optogenetic stimulation of the primary visual cortex drives activity in the visual association cortex
Source: Curr Res Neurobiol. 2023 Apr 8;4:100087. doi: 10.1016/j.crneur.2023.100087 (PMC10313868; doi:10.1016/j.crneur.2023.100087)
Supplement: Multimedia component 2 [file mmc2.docx]

Supplementary Material

**
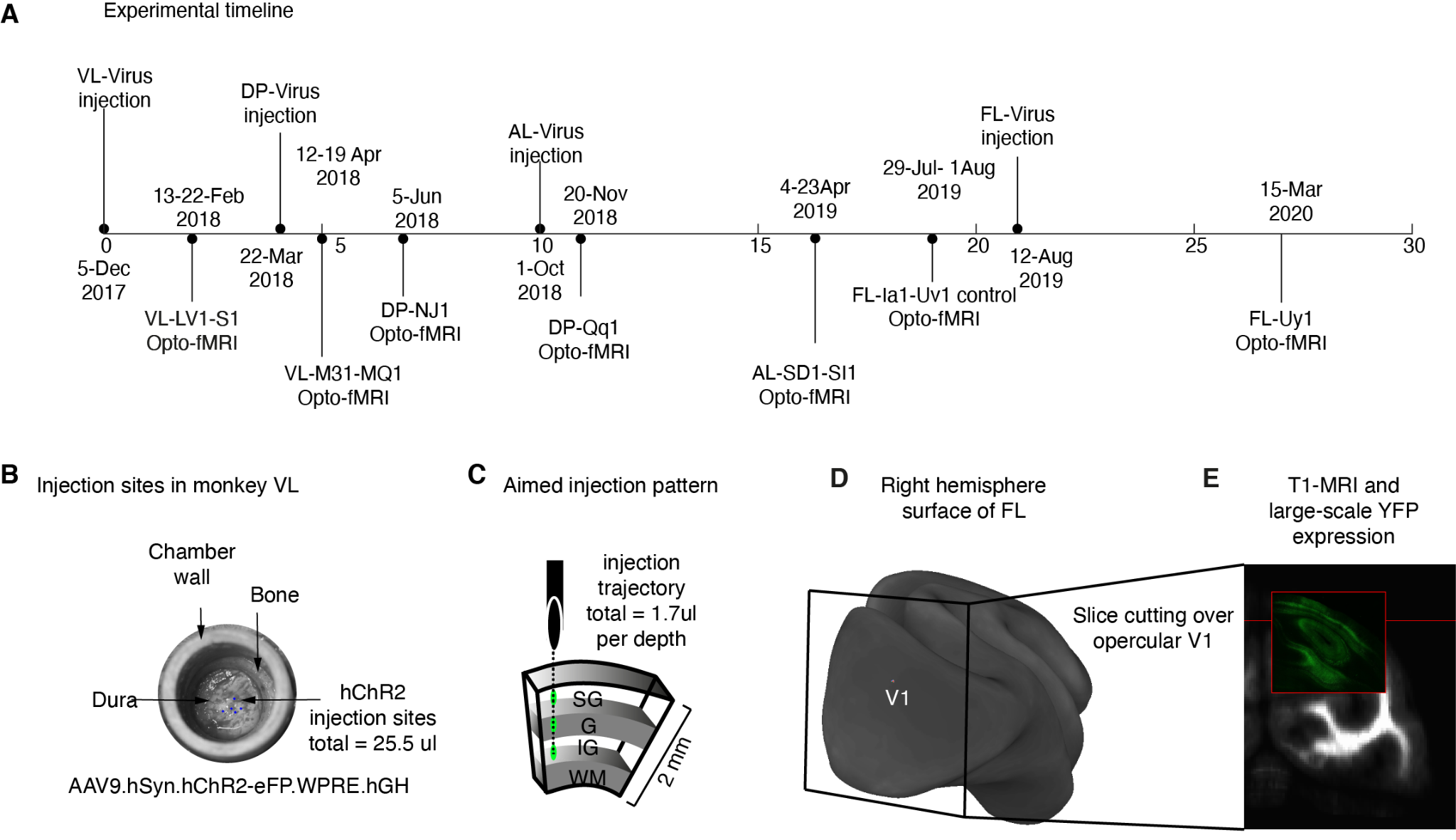
Supplementary Figure 1. Experimental timeline and injection approach**. **A**. Experimental timeline showing events for injections and opto-fMRI experimental sessions lasting 33 months. **B**. Photograph of chamber region with injection sites in monkey VL showing the cross-shaped injection pattern into cortical tissue. **C**. Illustration of the injection approach for each site with virus delivery at three different cortical depths with a total of 25.5*μl* of viral solution. **D**. Illustration of the V1 position of the slice showing eYFP expression in Figure 2B. **E.** Fluorescence of monkey FL overlaid to the corresponding coronal MRI section of the same monkey.

**
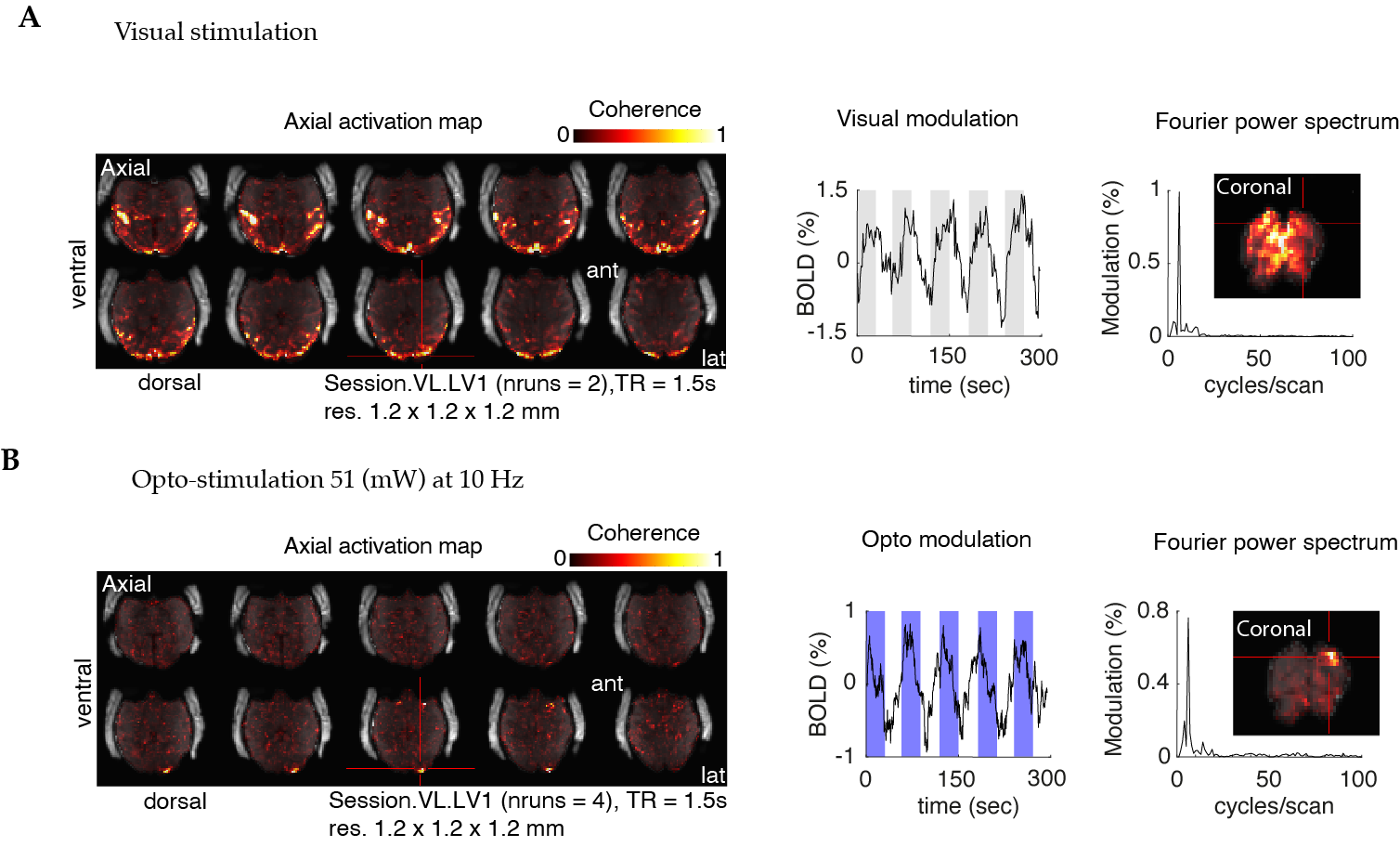
**

**Supplementary Figure 2**. **Comparison of V1 BOLD fMRI activation patterns in monkey VL elicited by optogenetic stimulation versus free-viewing of movie sequences.** **A**. Overall coherence map without threshold shows regions with significant BOLD activation in V1 and extrastriate cortex elicited by the free-viewing of natural scene movies. The middle panel shows the time course of BOLD signal modulation from an exemplary voxel in the recording chamber region. The right side panel shows the associated power spectrum with a peak at the stimulation frequency (0.016 Hz, 1/60 sec). The inset shows the coronal slice of the activation in the visual cortex. **B**. A similar plot as in A for the Opto-fMRI stimulation shows BOLD activation in response to optogenetic stimulation at 10Hz. Note that elicited activation is restricted to V1. Middle and right panels show BOLD signal modulation and power spectrum of the same V1 voxel with a modulation rate peak of 0.016 Hz or 1 cycle/60 sec. All data from monkey VL.

**
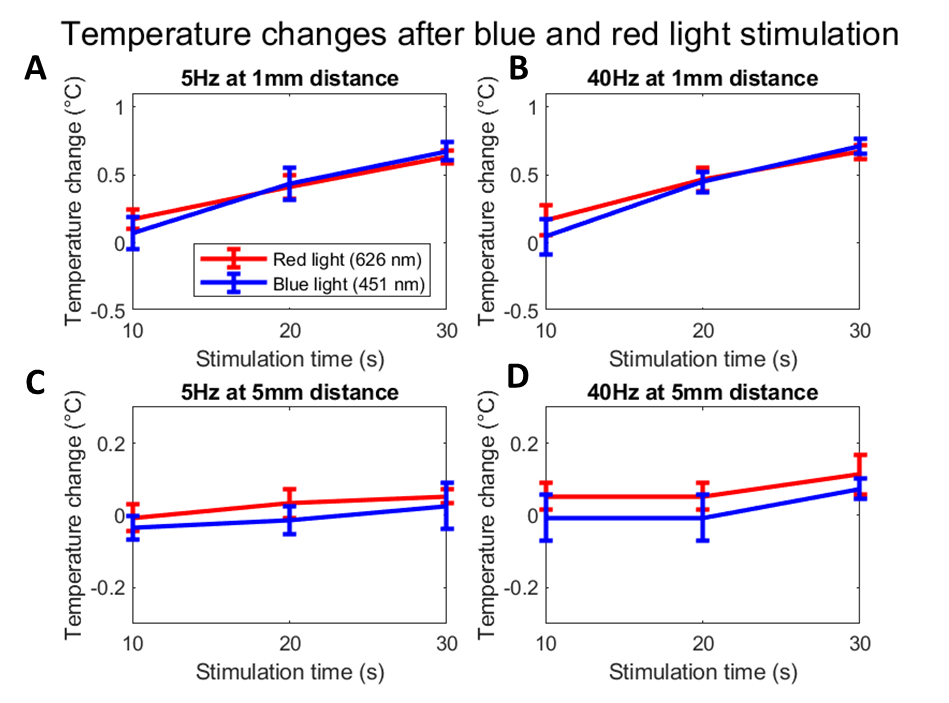
**

**Supplementary Figure 3. Mean temperature changes of ex vivo neuronal tissue in response to stimulation with blue and red light at 50mW over 30 seconds (5 repetitions).** A temperature probe was inserted into the neuronal tissue at two different distances from the surface of the electrode (1mm and 5mm). Temperatures were measured using a digital thermometer (Fluke 51). Temperature changes were calculated as differences from the baseline period during which there was no stimulation. **A.** Temperature changes measured inside the tissue at 1 mm distance from the LED after stimulation with blue and red light pulsed at 5Hz. **B.** Same as **A** but with a frequency of 40Hz. **C.** Temperature changes measured inside the tissue at 5 mm distance from the LED after stimulation with blue and red light at 5Hz. **D.** Same as **C** but with a frequency of 40Hz.. Note the y-axis scale difference between panels A & B vs C & D.

We observed an increase of tissue temperature of approx. 0.7°C over 30 seconds using both blue and red light at 5Hz (**A**, blue:0.67°C, red:0.63°C,40) and 40Hz (**B**, blue: 0.71°C red: 0.67°C) with a distance of 1 mm between LED and temperature probe. It is very likely that this increase in temperature was mostly caused by direct light from the LED rather than temperature increases of the tissue. To investigate this more closely, we increased the distance between LED and temperature probe to 5 mm and repeated the experiment. Here we observed only a marginal temperature increase by up to 0.1°C over 30 seconds using both red and blue light at 5Hz (**C**, blue: 0.03°C, red: 0.05°C) and 40Hz (**D**, blue: 0.07°C, red: 0.11°C) at a distance of 5mm between LED and temperature probe.

**
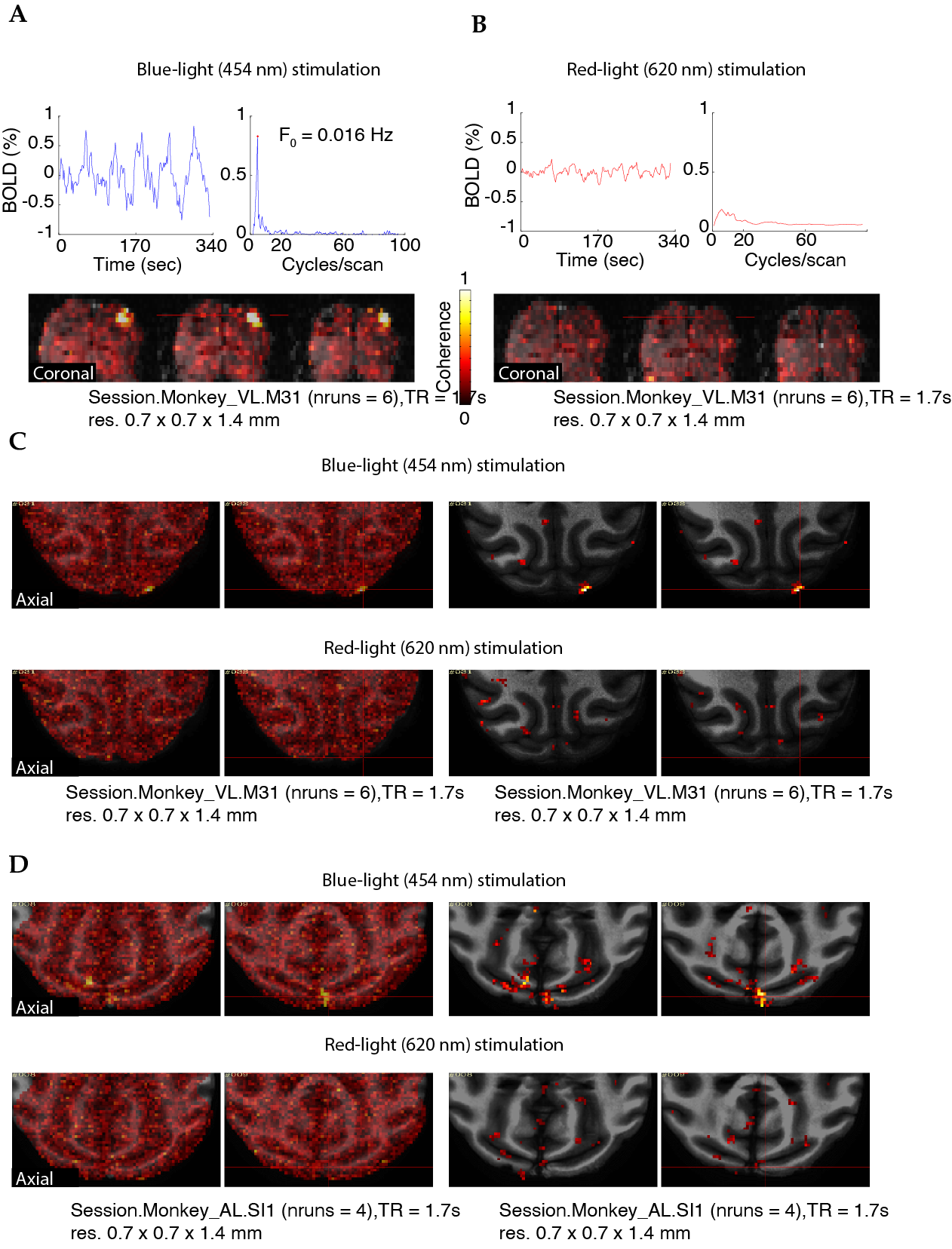
**

**Supplementary Figure 4**. **Wavelength specific stimulation drives the local positive BOLD response only after blue but not red-light stimulation.** **A**. Example voxel time course and power spectrum of BOLD signal modulation from blue-light (451 nm, 50 mW at 10Hz). Bottom panel shows the coherence map without a threshold of three coronal slices (n runs = 4) in monkey VL. **B**. Same voxel time course and power spectrum of BOLD signal modulation for monkey VL after red-light stimulation (626 nm, 50 mW at 10Hz). Note the absence of BOLD modulation under this stimulation condition. **C**. Shows the same coherence map as in B for monkey VL in the axial plane for both blue and red-light conditions. see coherence map without threshold. **D**. The coherence map without threshold for monkey AL in the axial plane for both blue and red-light conditions.

**
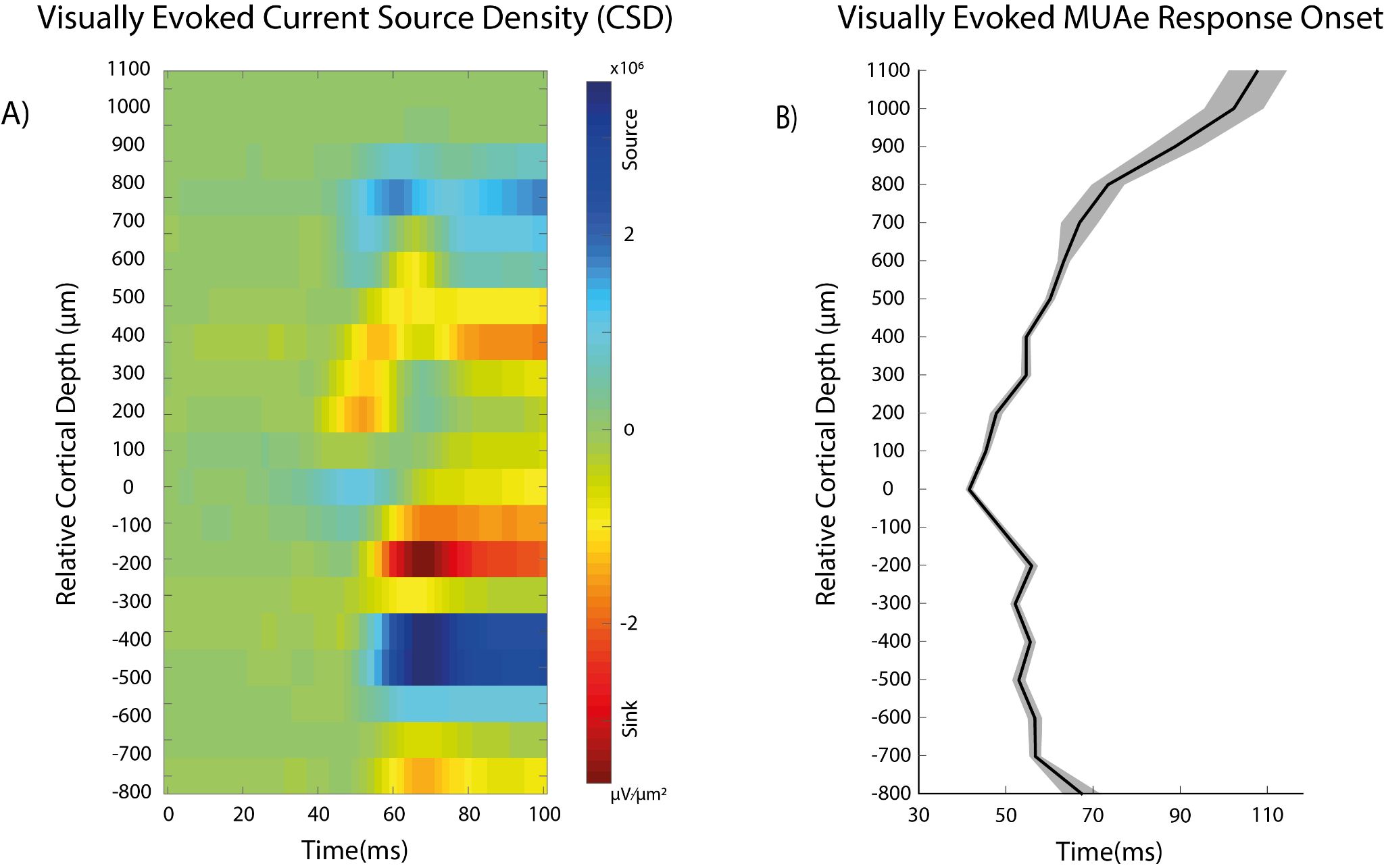
Supplementary Figure 5. Current Source Density (CSD) and latency of visually evoked MUAe responses in V1 as a function of relative cortical depth in monkey FL. A.** Example CSD response profile to a 5° patch of drifting gratings (spatial frequency 2 cycles/°, speed: 2-4 cycles/s) from one session. The lower border of the first sink (in red, thalamic input to L4C) is used as a reference to align laminar data across sessions. **B.** Response onset latency for the visually evoked MUAe across cortical depth across sessions. Latency onset was extracted when the signal crosses 4*SD of the baseline.

**
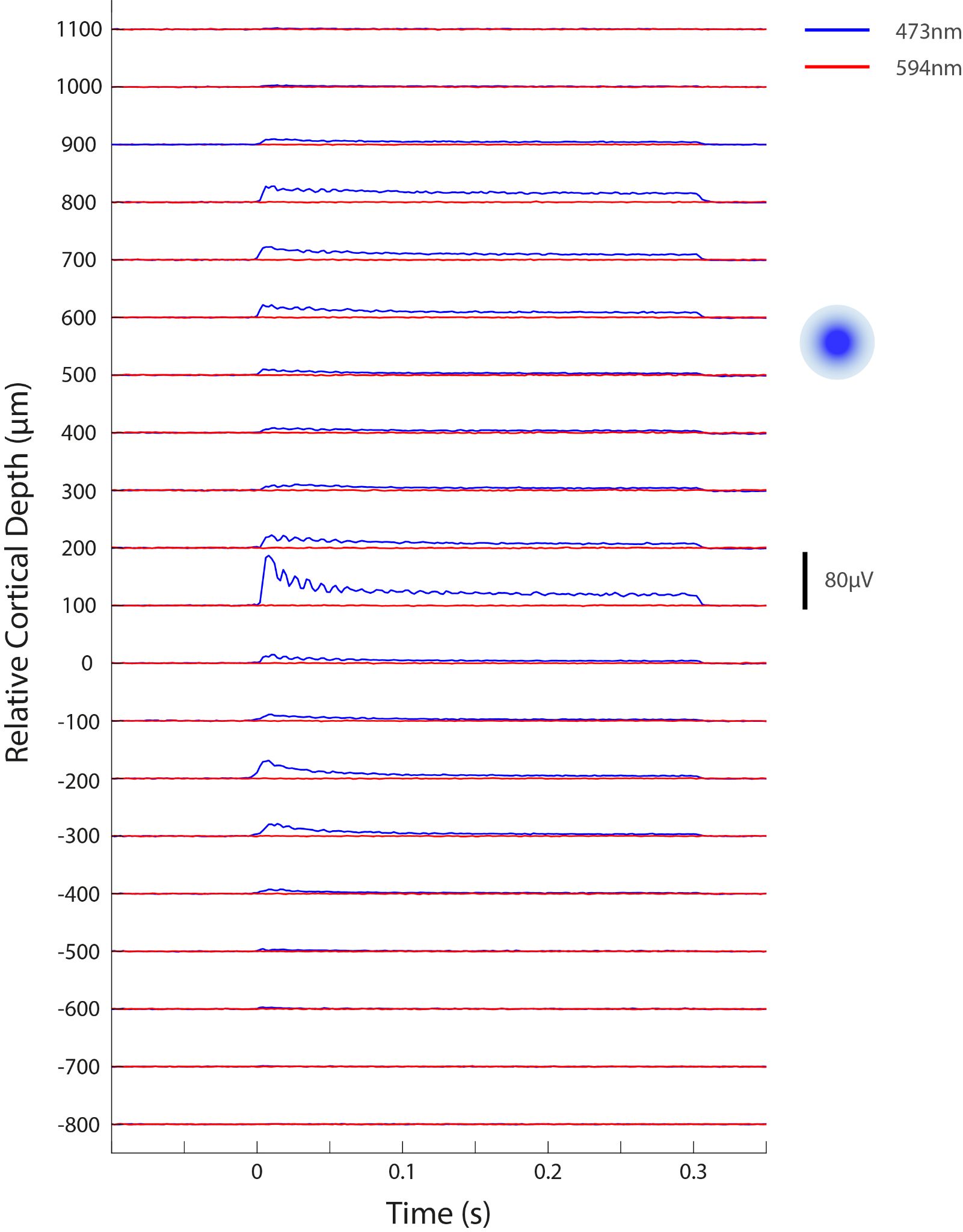
**

**Supplementary Figure 6. Example MUAe responses of monkey FL to both continuous blue (473 nm) and continuous red (594 nm) laser stimulation.** Red light stimulation (40mW) did not have an effect on neural activity (red) when compared to blue light stimulation with a similar power (37.8mW). The location of the embedded fibre is indicated by the blue circle on the right.


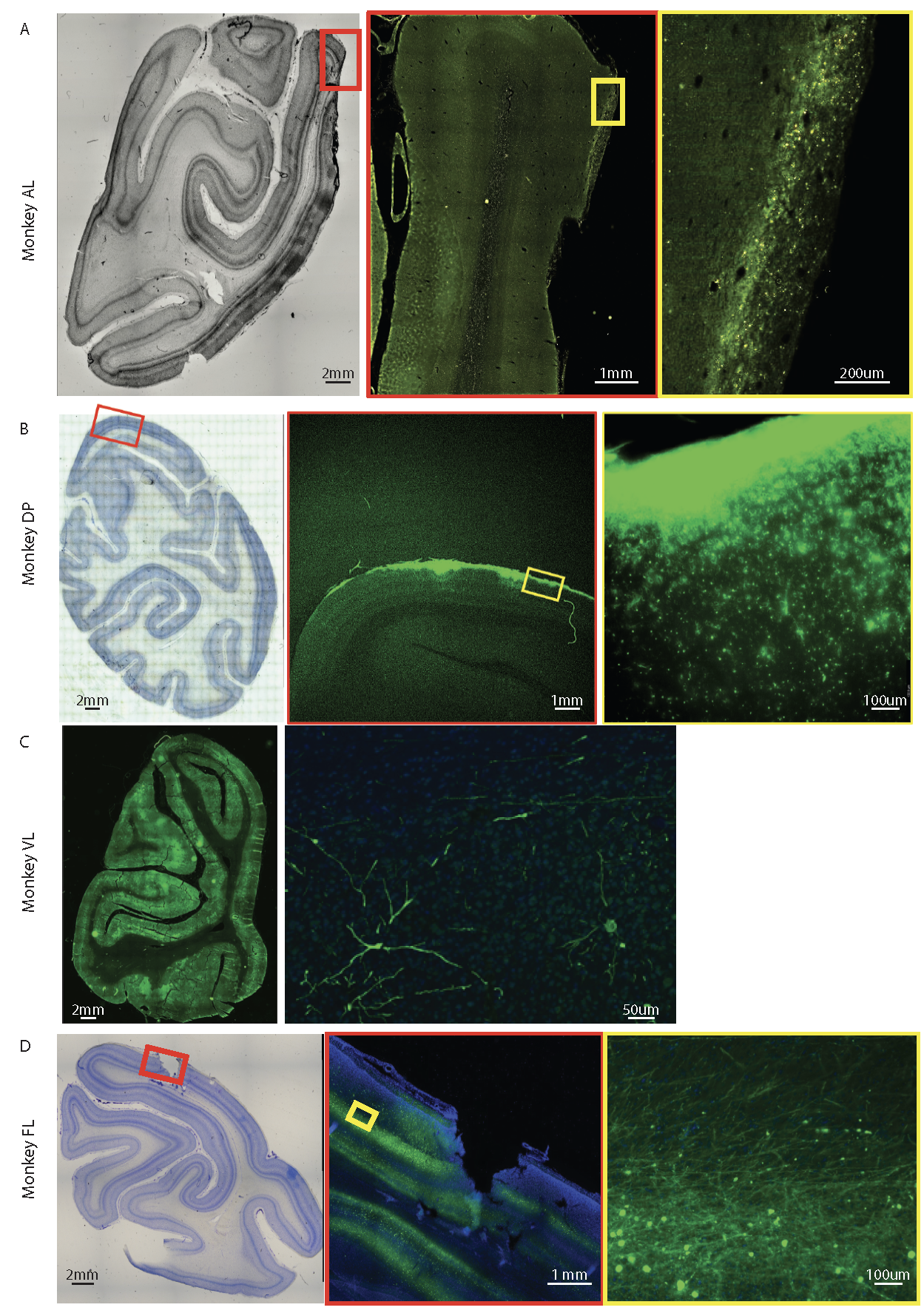


**Supplementary Figure 7*.*** AAV9-hsyn-Chr2-eYFP expression in the four participating monkeys. **A**. Expression pattern of monkey AL. Nissl stained brain slice indicating area V1 (left, red rectangle). Fluorescence of AAV construct (bright green) in V1 (middle). Magnification of V1 showing expression of AAV construct (bright green, right). **B**. Expression pattern of monkey DP. Nissl stained brain slice indicating area V1 (left, red rectangle). Fluorescence of AAV construct (bright green) in V1 (middle). Magnification of V1 showing expression of AAV construct (bright green, right). **C**. Expression pattern of monkey VL. Fluorescence image of brain slice indicating area V1 (left, red rectangle). Magnification of V1 showing somatic and dendritic expression of AAV construct in V1 (right). **D**. Expression pattern of monkey Fl. Nissl stained brain slice indicating area V1 (left, red rectangle). Fluorescence of AAV construct (bright green) in V1 (middle) co-stained with the somatic marker DAPI (blue) showing laminar distributing pattern (see Figure 2 for details). Magnification of V1 showing somatic and dendritic expression of AAV construct (bright green, right).

**
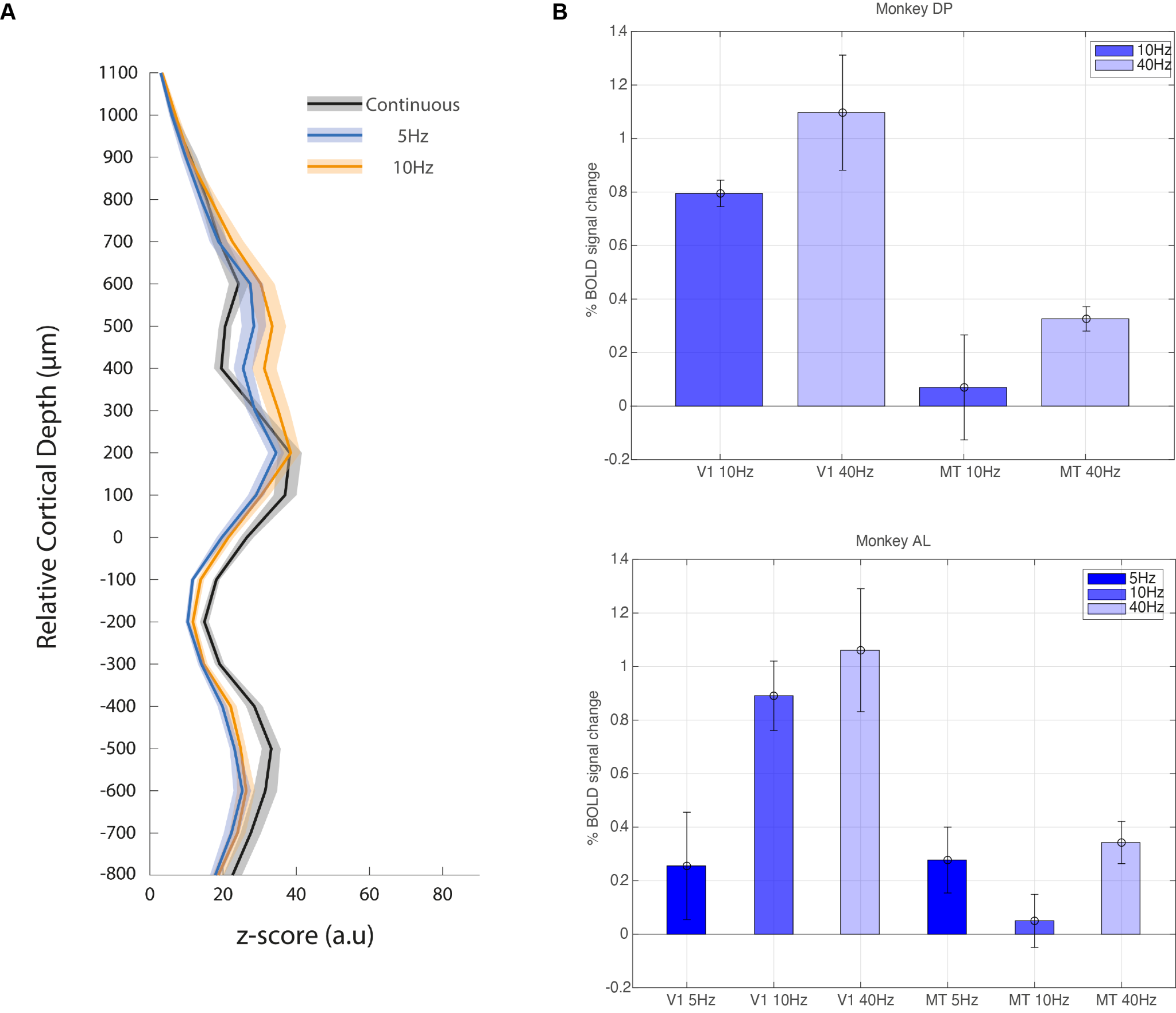
**

**Supplementary Figure 8. Laminar and BOLD activation for optogenetic multiple stimulation frequencies. A.** Similar to Fig. 3, the laminar activation pattern is calculated for continuous (black) stimulation as well as 5Hz (blue) and 10Hz (orange) stimulation frequencies. The average MUAe firing rates are calculated for 100-300ms post stimulation onset for the continuous stimulation and 100-900ms post the first stimulation pulse onset for the pulsed stimulation. **B**. Average percent BOLD signal change of the V1/V2 local cluster region of monkey AL tested at three different frequency levels (5 Hz, 10 Hz and 40 Hz) and on monkey DP tested at two different stimulation frequencies (10Hz and 40Hz).

***
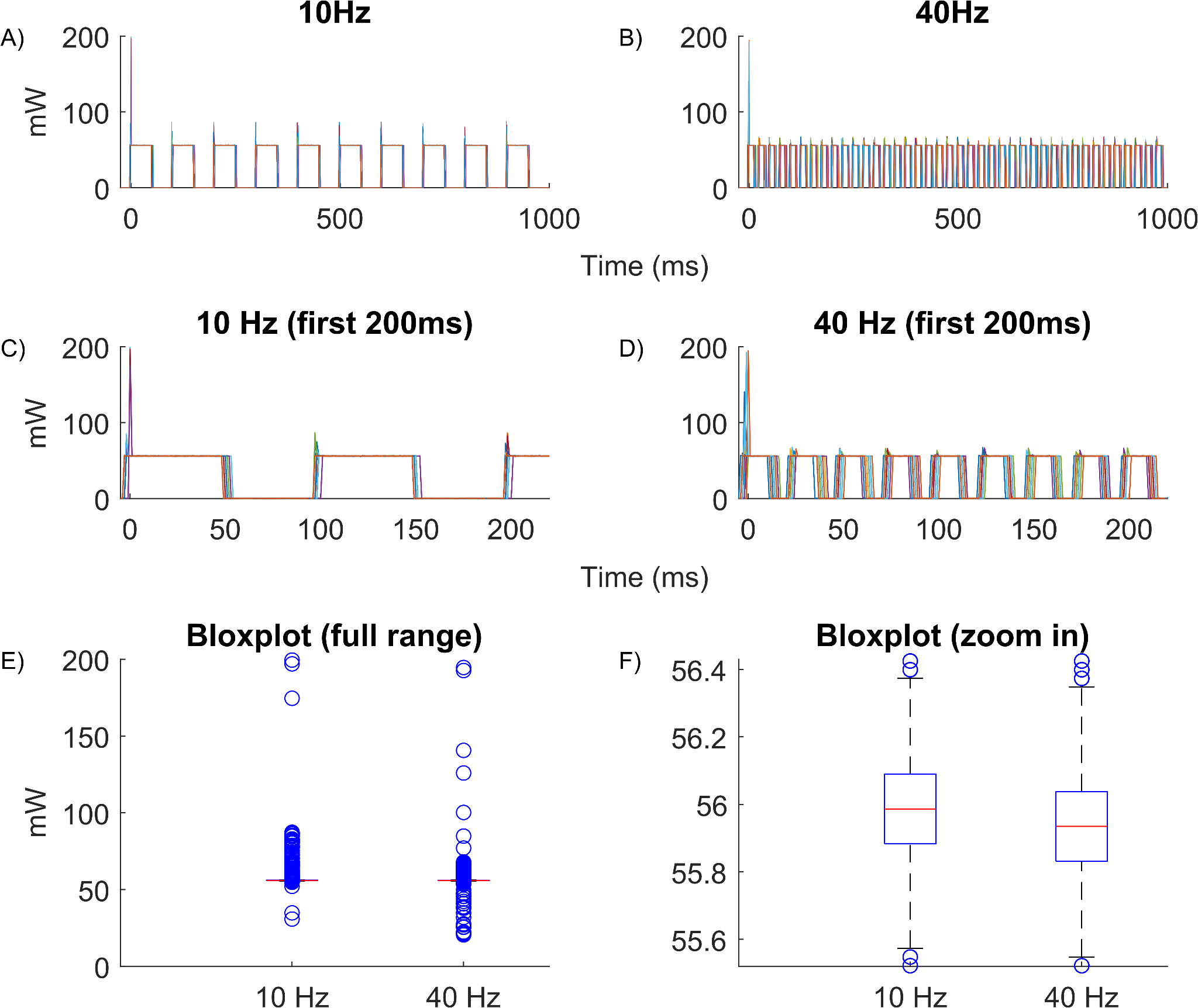
***

**Supplementary Figure 9. Measurement of LED output for 10 Hz vs 40 Hz stimulation frequencies.** Power generated by the 451nm LED stimulator was measured by connecting an optic fibre from the output of the stimulator to an integrating sphere photodiode power sensor (s145 Thorlabs). Readings were taken and transformed to current (PM100D Thorlabs), output connected to a Cerebus recording system (Blackrock Neurotech) and digitised at 1 kHz. The stimulation protocol consisted of 1 second of ON activity (LED on), followed by 1.5 seconds OFF period. The alternating sequence of pulses lasted around 2 minutes (approximately 100 ON sequences per condition). LED power was fixed at 56 mW. With this protocol, two sequences of pulses were generated, one with a frequency of 10 Hz and another with 40 Hz.

**A, B** Time series for the 10 vs 40 Hz stimulation runs (here, traces have been overlaid for comparison purposes). Although there are some power spikes for both protocols, the spikes are brief and the average amplitude is steady over time. The spikes represent transient power spikes exceeding the intended 56 mW power modulation. These power spikes could reach up to 200 mW when the LED was turned on for the first time following a longer period of no light emission. Their amplitude decreased rapidly to a transient level around 70 mW for repeated stimulation epochs. **C, D** Time series for the first 200 ms of the stimulation epochs from **A** and **B** since the larger power spikes were typically observed at the beginning of the stimulation epochs. Importantly, the duration of the spikes did not exceed a few milliseconds, i.e. much shorter than the stimulation periods (30 seconds of stimulation) used for the fMRI experiments and the TR of the EPI sequence.

In order to quantify the power for both conditions, we measured the power across all ON periods for both stimulation conditions. **E** An overview over the power distributions with the transients clearly visible. **F.** A more zoomed in version of the distribution in **E** focusing on the median and interquartile range. From this analysis, it can be concluded that although transient activity is present in both 10 Hz and 40 Hz conditions, that does not lead to more power delivered when 40Hz frequency is used since the median power output is practically the same (10 Hz: 55.98 mW; 40 Hz: 55.93 mW) for both conditions. The differences observed in area MT fMRI activity (Figure 3 of the manuscript) are therefore attributed to frequency manipulation rather than a difference in power delivery resulting from the inclusion of more power transients.


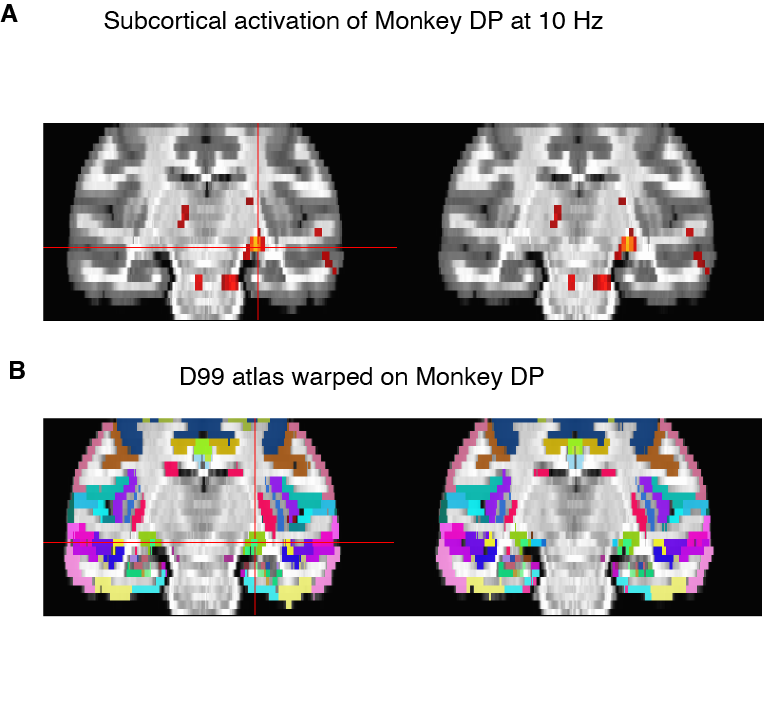


**Supplementary Figure 10. Extrastriate and subcortical activation maps in monkey DP.** Similar to **Fig. 3E**, **F**. **A**. Two coronal slices with significant activation of the lateral geniculate nucleus (LGN) of the stimulated hemisphere. **B**. Regions from the D99 atlas mapped onto the warped template of monkey DP.


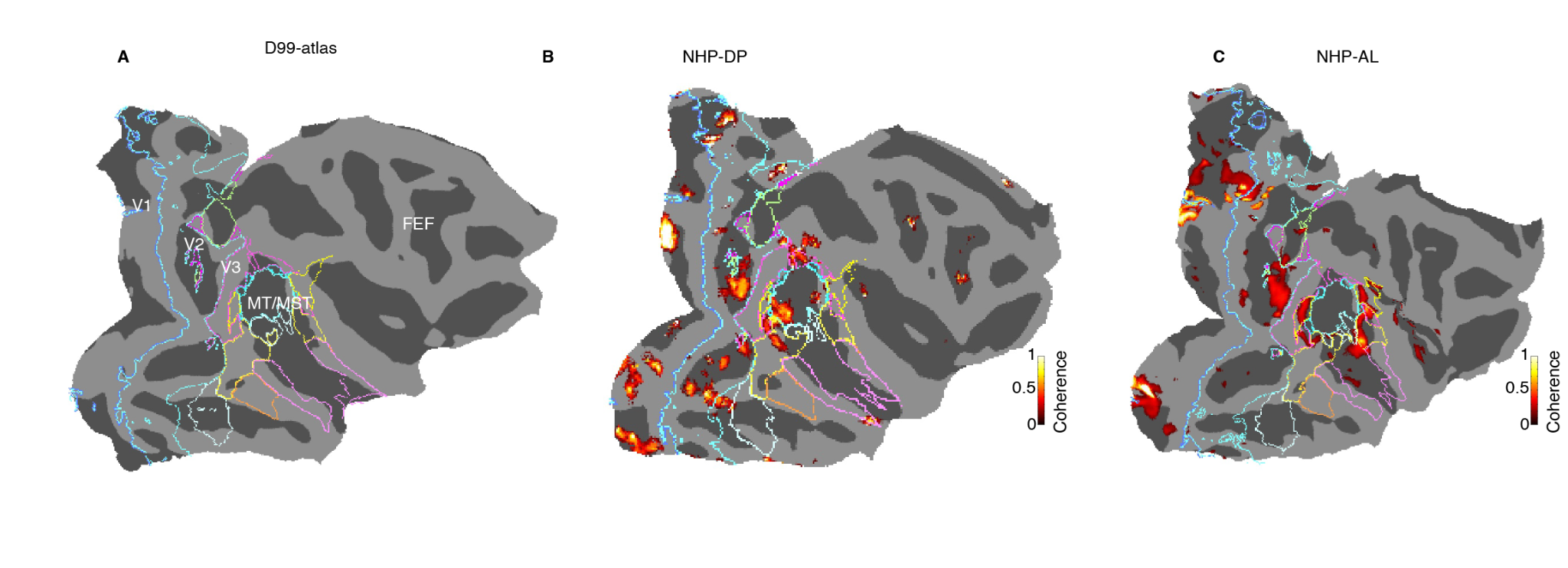


**Supplementary Figure 11. V1 optogenetic stimulation drives BOLD activation in higher cortical areas**. **A**. Flat map of D99-atlas showing the overall cortical parcellation for the average brain map. Activation maps showing extrastriate BOLD activity in V1, V2, V3 and motion complex regions MT/MST in Monkeys DP (**B**) and AL (**C**).


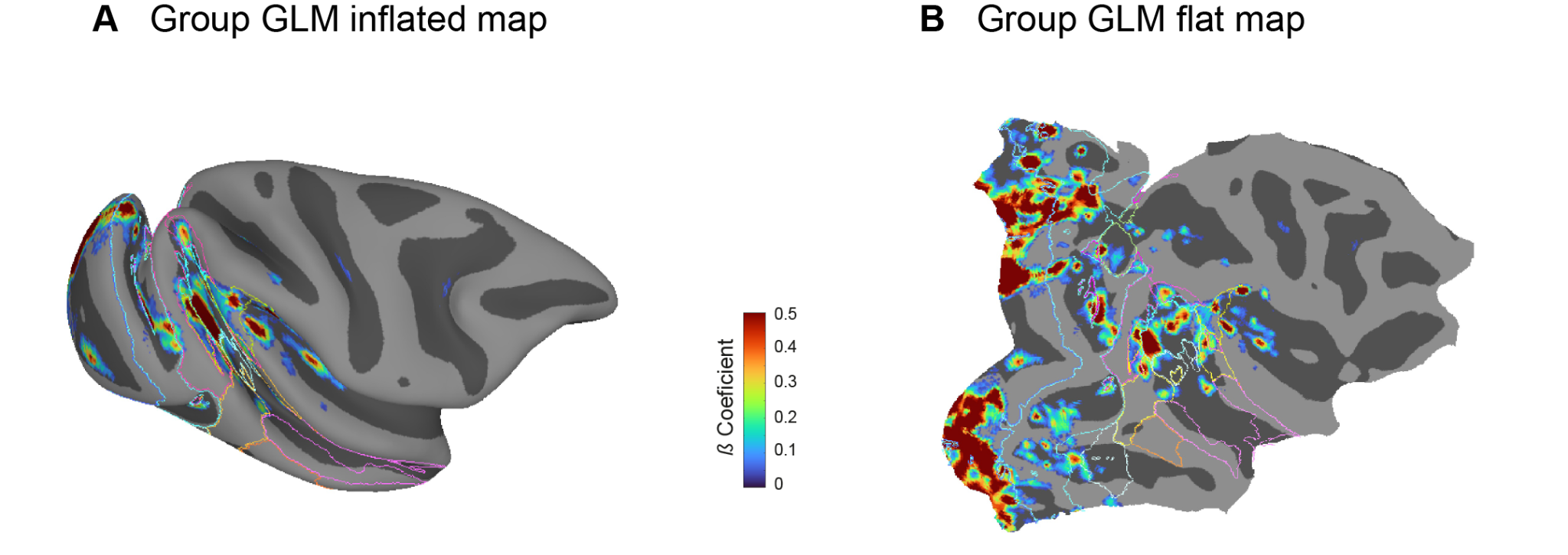


**Supplementary Figure 12. General linear model analyses.** As the input dataset to 3dDeconvolve, we provided the detrended time-series and then applied ordinary least squares to a regression model of the BOLD signal which included a gamma variate block design of the hemodynamic response to the opto-stimulation periods (30 secs on and off). A general linear T-tests were performed between opto-stimulation and the no stimulation periods. The stats output from 3dDeconvolve was then used to visualise the activation areas. The threshold was chosen to be at a significant p-value < 0.01. We confirmed significant activation (T-value colormap range 1.9 < 8.3) across visual and dorsal stream related regions in the average map. Maps are displayed on the inflated surface (**A**) and the flat map surface (**B**).


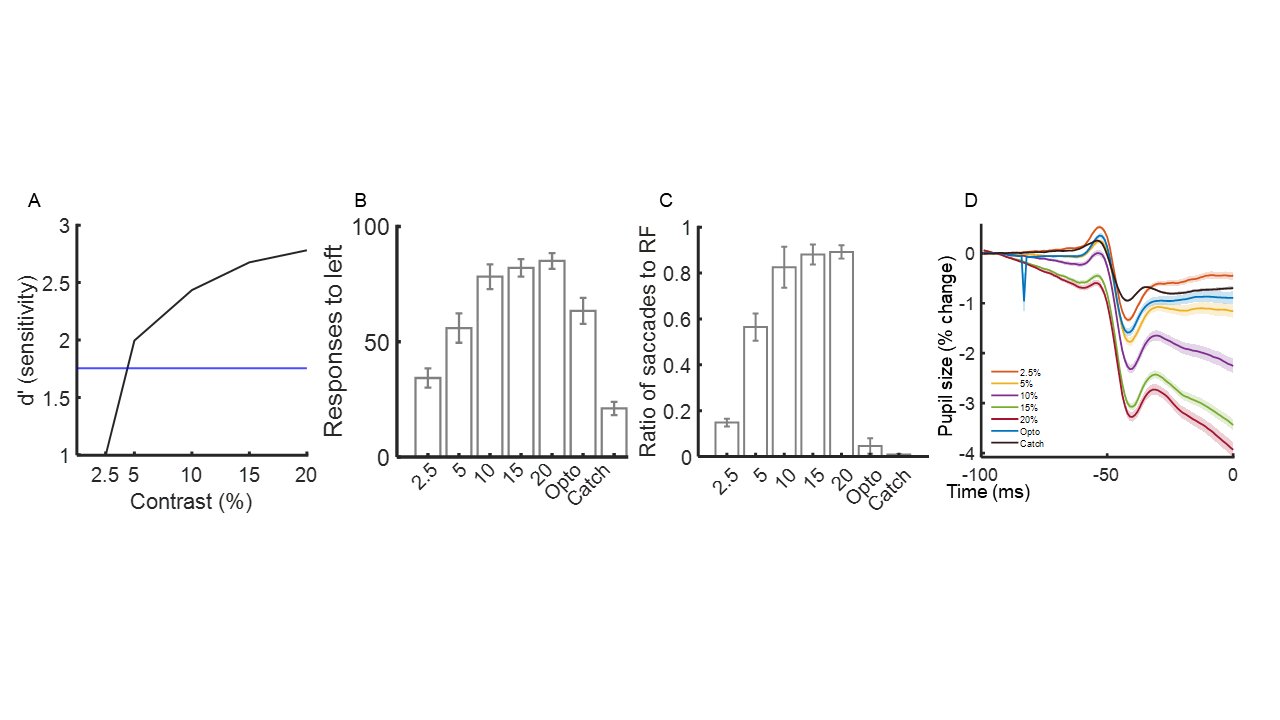


**Supplementary Figure 13.** **Behavioural confirmation of phosphene perception**. A) Task performance on the first day of optogenetic testing following previous visual training. The monkey generalised immediately from visual to optogenetic task performance. For further information and performance across sessions, see Fig. 5. B) Proportion of saccades (direct and indirect via RF) to the left ‘yes’ target across conditions and experiments. Note that the monkey made a false positive response in only approx. 20% of catch trials, contrasting with the much higher hit rate during visual and optogenetics trials. C) Ratio of initial saccades to the receptive field (RF) during ‘yes’ trials to the left response target. D) Pupil size changes before (-100ms to 0ms) the first saccadic response for correct trials across conditions. Increasing visual luminance increased pupil constriction and pupil restriction to optogenetic stimulation was similar to that of visual stimuli with lower luminance.

**Table 1**

|  | ***Blue light*** |  | ***Red light*** |  | ***Movie*** |  |  |
| --- | --- | --- | --- | --- | --- | --- | --- |
| ***Monkey*** | **Activation cluster mean coherence** | **Activation cluster sigma coherence** | **Activation cluster mean coherence** | **Activation cluster sigma coherence** | **Activation cluster mean coherence** | **Activation cluster sigma coherence** |  |
| *VL* | 0.4 | 0.2 | 0.14 | 0.08 | 0.62 | 0.19 |  |
| *DP* | 0.55 | 0.28 | - | - | 0.33 | 0.18 |  |
| *AL* | 0.45 | 0.16 | 0.21 | 0.09 | 0.31 | 0.09 |  |
|  |  |  |  |  |  |  |  |
|  | **Blue light** |  | **Red light** |  | **Movie** |  |  |
| ***Monkey*** | **Activation cluster mean phase** | **Activation cluster sigma phase** | **Activation cluster mean phase** | **Activation cluster sigma phase** | **Activation cluster mean phase** | **Activation cluster sigma phase** |  |
| *VL* | 196.78 | 30.62 | 151.53 | 44.2 | 172.33 | 35.62 |  |
| *DP* | 150.22 | 23.15 | - | - | 147.95 | 24.46 |  |
| *AL* | 103.23 | 26.81 | 131.2 | 35.37 | 214.91 | 39.83 |  |
|  |  |  |  |  |  |  |  |
| ***Monkey*** | **Total number active voxels** | **Total number of V1 voxels** | **V1 volume (mm3)** | **Percent activation of V1 volume** | **Activation volume (mm3)** | **voxel size mm3** |  |
| *VL* | 1840 | 72199 | 1949.37 | 2.55% | 49.68 | 0.3 |  |
| *DP* | 2816 | 71383 | 1927.34 | 3.94% | 76.03 | 0.3 |  |
| *AL* | 3709 | 83422 | 2252.4 | 4.45% | 100.43 | 0.3 |  |
|  |  |  |  |  |  |  |  |
| ***Monkey*** | **Total number active surface voxels** | **Total number of surface V1 voxels** | **V1 area (mm2)** | **Percent activation of V1 area** | **Activation area (mm2)** | **voxel size mm2** |  |
| *AL* | 535 | 11493 | 1034.27 | 4.65% | 48.15 | 0.3 x 0.3 |  |
| *DP* | 808 | 11401 | 1026.09 | 7.08% | 72.72 | 0.3 x 0.3 |  |
| *AL* | 1354 | 12585 | 1132.65 | 10.75% | 121.86 | 0.3 x 0.3 |  |

| ***Monkey*** | **Activation cluster mean %CNR** | **Activation cluster sigma %CNR** | **Activation cluster mean %CNR** | **Activation cluster sigma %CNR** | **Activation cluster mean %CNR** | **Activation cluster sigma %CNR** |
| --- | --- | --- | --- | --- | --- | --- |
| *VL* | 0.7 | 0.1 | 0.4 | 0.2 | 2.0 | 0.12 |
| *DP* | 0.76 | 0.14 | - | - | 1.6 | 0.4 |
| *AL* | 0.94 | 0.2 | 0.05 | 0.1 | - | - |
