# Supplementary material for: Optogenetic stimulation of the primary visual cortex drives activity in the visual association cortex
Source: Curr Res Neurobiol. 2023 Apr 8;4:100087. doi: 10.1016/j.crneur.2023.100087 (PMC10313868; doi:10.1016/j.crneur.2023.100087)
Supplement: Multimedia component 1 [file mmc1.pdf]

Peer Review Overview

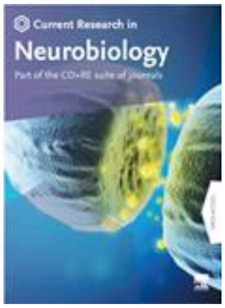

Manuscript Title: Optogenetic stimulation of the primary visual cortex drives activity in the visual association cortex

Received	Aug 26, 2022
1st Decision	Oct 14, 2022
1st Revision Submitted	Feb 14, 2023
Accepted	Mar 22, 2023

1st Decision letter

Reference: CRNEUR-D-22-00114
Title: Optogenetic stimulation of the primary visual cortex drives activity in the visual association cortex
Journal: Current Research in Neurobiology

Dear Prof Schmid,

Thank you for submitting your manuscript to Current Research in Neurobiology.

I have completed my evaluation of your manuscript. The reviewers recommend reconsideration of your manuscript following major revision. I invite you to resubmit your manuscript after addressing the comments below. Please resubmit your revised manuscript by Dec 13, 2022.

When revising your manuscript, please consider all issues mentioned in the reviewers' comments carefully: please outline every change made in response to their comments and provide suitable rebuttals for any comments not addressed. Please note that your revised submission may need to be re-reviewed.

Current Research in Neurobiology values your contribution and I look forward to receiving your revised manuscript.

Kind regards,

Yogita Chudasama
Associate Editor
Current Research in Neurobiology

Comments from Editors and Reviewers:

Reviewer #1:

This paper reports an interesting set of experiments in macaques examining the effect of optogenetic stimulation of V1 in other brain regions (using fMRI) and on behavior (but only in one monkey subject). The authors find that optogenetic stimulation of V1 results in activation of dorsal but not ventral stream extrastriate areas. They relate this to the laminar expression pattern of the opsin, which was predominantly in layer 4B, which projects to MT. Furthermore, they report behavioral data of one monkey showing perceptual detection of the optogenetic stimulation.
This is a well-conducted study, combining optogenetics, histology, electrophysiology, fMRI and behavior. I do not have major technical or methodological concerns but have suggestions to improve the manuscript.
Main comments.
1. It was not clear to me what the monkeys were doing during the fMRI scanning. Were they performing a passive fixation task? Any behavioral control? Behavioral state: were they fully awake? Was there a difference in behavior between the stimulation, i.e. stimulus frequency, conditions? Please clarify.
2. They state that the LED delivered light up to 3.5 mm in depth. It appears that they based this on experiments using agar. However, did they consider that blue light penetrates less deep into the brain?
3. Although the expression of the opsin is stronger in layer 4B than in other layers, the 40Hz photostimulation produced strong MUA in layer 3. The latter layer project to V2 and then ventral visual stream areas. Hence, it is not clear why such strong responses in layer 3 did not activate ventral visual stream areas. I think given the discrepancy between the 40Hz electrophysiology data and the histology it is too simplistic to explain the specific dorsal stream activations by the laminar expression pattern of the opsin. This should be discussed in greater detail and in a more nuanced fashion.
4. Figure 4 appears to show activations due to photostimulation in the STS anterior to MT/MST. Are these significant? Can these be shown on coronal sections? Were they present in both banks of the STS?
5. The behavioral data are interesting but somewhat preliminary because they involve only one animal. Furthermore, behavioral effects of V1 optogenetic stimulation in monkeys has been shown before (e.g. recent report by Chen et al., Elife, 2022; which the author should cite and discuss). The authors claim that the monkeys perceive the optogenetic stimulus as a visual stimulus of 5-10% contrast. This is based on the psycho- and chronometric curves of Figure 5C and D. However, the data shown in Figure 5B suggests that even a 5%, and to some extent a 2.5 % contrast visual stimulus produces a difference in behavioral responses to the target compared to the photostimulation. This discrepancy between the two behavioral measures (d' and saccade to target) with respect to the comparison between photostimulation and visual stimulation should be addressed.
Minor comments.
1. The authors show no response to red light stimulation, which they use as a control for heating and other artefacts. However, red light heats neural tissue less than blue light. It would have been better to use as a control stimulation by blue light of a non-injected part of the visual cortex. Nonetheless, it is unlikely that the effects - fMRI and electrophysiology - are due to heating and/or artefacts.
2. Eye movement analysis: "We detected eye-movements automatically with a usually employed velocity based algorithm". What is the "usually" employed velocity algorithm?
3. Figure 3: it would be nice to show the exact timing of the stimulation blocks at the top of A.

Reviewer #2:

In this paper titled "Optogenetic stimulation of the primary visual cortex selectively drives cortical dorsal stream activity", Ortiz-Rios et al. use optical fibers combined with laminar recording and fMRI in macaque monkeys in order to delineate the functional connectivity of the areas immediately after V1 in the dorsal visual hierarchy. In one monkey, they go beyond and perform a behavioral experiment and demonstrate the effect of optogenetic stimulation of V1 on a psychophysical task, as well as the eye movements and the pupil response.

In general, I find the study carefully designed and meticulously performed. The central question of the paper is of utmost importance in the field. From a technical point of view, the study is impressive given the significant challenges of combining optogenetics with fMRI, neural recordings and behavioral testing in nonhuman primates. Nevertheless, I have some critical concerns, addressing which would increase the quality of the paper.

Major concerns:

1. Red light photons carry ~1.8 electron volts of energy while the blue light photons are in the range 3.1 eV. I am not sure if the energy levels (range of 50mW) reported in the paper are photometric powers measured out of the optical fiber or electrical power levels used to drive the LEDs? In other words, I don't know if the red (control) vs blue (test) powers are equated for the total photo power that was injected into the tissue or for the electrical power that was used to drive the LEDs? If the former, then the electrical power must have been higher for the red LEDs to compensate for the weaker photons (when heating is to be controlled), is that true?

2. On a very related and perhaps more important point, long wavelength light is absorbed in the body tissue and blood far less than the short wavelengths. This can be easily demonstrated by covering the tip of red and green laser pointers with one's thumb; the green doesn't go through but the red illuminates the entire thumb. This in fact is the reason for developing red-shifted opsins in order to allow further light penetration in the tissue. Further penetration means less absorption and less heating, this jeopardizes the red light as a control for heating, which is a serious concern here given the long durations of optical stimulation. The authors are probably aware of this issue as they have mentioned in the paper that the ideal control experiment here is using the blue light on intact neural tissue (not transduced). I don't have the numbers, but if the absorption of the red light in a given volume of tissue around the fiber tip is less than the blue light by less than 1 order of magnitude, it could serve as a partial control as it would be possible to estimate the upper bound of a possible heating effect. But I doubt if that is true. Either way, I believe this shortcoming should be mentioned and addressed in the paper. I agree with the analysis of the authors that the sensitivity of the observed effect to temporal frequencies at relatively high rates cannot be explained with heating given its sluggish temporal response. I think this point should be inflated, the point about the red being a control, deflated.

3. The animals were first trained with a visual stimulus, then they entered a 3 conditioned task that included 33.3% brain stimulation trials. It is possible to argue that the animals did not perceive the optogenetic stimulation, but their brain circuit rewired and they learned a new rule to perform this new class of trials bypassing visual perception. Showing the performance of the first day when cortical stimulation is introduced can be helpful in this regard. Did the monkey perform the task at chance for the optogenetic trials in the first day then gradually learned it over the course of multiple days? Or was there above chance performance even in the first day? That would imply easy generalization of the

visual task to the optogenetic task and help the discussion specially in the absence of strong eye movement evidence in support of the phosphene theory.

4. The design feature that allows free eye movements before landing at a response targets is brilliant. Its results though need to be more clearly communicated. Specifically, it is not clear what happens to the saccadic eye movements when the optogenetic stimulation is applied. The authors show that saccades to the RF are less common than the high contrast stimulus and not different from the low contrast stimulus, but are they different from the no stimulus condition? Figure 5 A shows a trace of an effect, but I failed to find a quantitative analysis of that small effect. The question is basically: is there any significant bias for saccading to the RF? Also see my minor comment #2. Related comment: my hat off to the amazing pupil effect.

Minor concerns:

1. Technically, the behavioral task used here is not a 2AFC. Apart from having 2 alternatives, what is referred to as the two alternative forced choice design is a specific design (not necessarily good for this study) that aims at zeroing the effect of choice biases. In a 2AFC, both stimulus conditions are presented in each trial (side by side or in temporal sequence) and the subject is asked to choose which one (left or right, first or second, etc.) contained a given signal (eg brain stimulation). In this way, even if the animal constantly chooses one response target, it will receive only 50% reward. The design used here is not 2AFC as it only has one stimulus in each trial and its chance level vary based on choice bias. Note that if the monkey chooses to pick the left target all the time he will receive 66% reward, but if he picks the right target all the time he will receive only 33%. It is OK as d' is used here for measuring performance, but the task is not 2AFC.

2. Subplot 5.b shows the ratio of direct saccades to the target, but it would be more informative to plot the saccades to the target/RF.

3. In the discussion, in the review of optogenetic studies regarding V1 and phosphenes Rajalingham et al., 2021, Nature Methods is missing. There, using an optical array we have shown that optogenetic silencing in V1 induces transient scotomas in the corresponding part of the visual field in the form of zones of elevated threshold in a direct contrast comparison task between two targets. The results are very related to this study and I am curious to see how the effect sizes reported here compare to the opposite direction effect sizes from silencing.

4. In the introduction the primate brains are referred to as "more evolved" in comparison to rodent brains. Well, throughout the human history we have enjoyed putting ourselves on top of an evolutionary ladder and I don't mind the authors sitting there, but I humbly bring it to attention that evolution doesn't have a direction, it is an ever changing network and our street rats are as "evolved" (whatever that term means) as us, but in other dimensions. I would prefer a more objective term like: large primate brains, larger and more complex primate brains, etc.

Reviewer #3:

This study describes an exciting application of the cutting-edge optogenetic-fMRI technology to reveal brain wide cell-specific connections in non-human primates (NHP). Although application of optogenetic stimulation mapping brain functional networks has been conducted in several animal model, applying optogenetic methods to primate brain is challenging. Thus far, only a few studies successfully to

manipulate behavior in NHP using optogenetics. Here, the authors demonstrated a large-scale circuit connection induced by optogenetic stimulation using fMRI. To confirm the effectiveness of the stimulation, multiple methods including electrophysiology, immunohistochemistry and behavior were applied. The succeed in achieving optogenetic manipulation and network mapping in macaque provide referential value for the neuroscience community and highly benefit translational neuroscience. Details of the study were comprehensively described and statistical analyses were properly performed. I only have three major concerns and some minor comments as detailed below.

Major concern:
1. The definition of connected site location is suspected. Although the activation location of MT in volume view is convincing (Fig. 3 D), the activations on surface view seems have a mismatch (Fig. 4 E). The MT/MST is located in the superior temporal sulcus. However, there is an obvious connected site located in the gyrus between lunate sulcus (LS) and superior temporal sulcus (STS) where seems to be V4. This is also true in Supp. Fig. 10 and graphical abstract.
In addition, there are also other areas been activated beside V2, V3 and MT/MST. For example, in figure 4 E, there are highly activated clusters in the Postcentral gyrus (maybe somatosensory cortex) and anterior end of temporal cortex (maybe TEa). The authors only describe these areas in dorsal stream but neglect of other areas which is too subjective.
Moreover, V2 has different compartments selective to either ventral stream or dorsal stream such as "color" stripes and "motion" stripes. The activation map here can't define which compartment they are. With saying this, the hypothesis that "optogenetics induced projection along the cortical dorsal stream" is also questioned, because V1 layer 4 also been reported to selective to orientation. Orientation domains were also detected in ventral stream areas like V4.
In general, from both the results and hypothesis side, the conclusion of "selectively drives cortical dorsal stream activity" looks overstated.

For better understanding of these maps, the name of the important sulcus (e.g., LS and STS) should be labeled as a marker of geometry. Please also indicate the color indices of gyrus and sulcus. I assume the surface follows Freesurfer convention that dark gray is sulcus and light gray is gyrus. It would also be helpful to only keep the D99 atlas borders that related to the activations in this study. The current figure, with so many areas, is not easy for readers to localize the activations.

2. The rational to use the coherence analysis mentioned by authors is "the hemodynamic response function (HRF) of opto-fMRI in macaques has not been formally characterized". This rational doesn't sound strong to me because the block design used in the fMRI study should be less influenced by HRF. Although the coherence analysis has been used in many studies, the GLM analysis method is still the most conventional method to detect activations. As probably been one of the first few papers to show optogenetic induce brain connections in MRI, I suggest author also show the activation map from conventional GLM analysis, at least in supplemental. From methodological view this will be help to boost further opto-fMRI studies and the understanding of optogenetic BOLD physiology.

3. What's the level of reproducibility of these large-scale cortical circuits induced by optogenetic stimulation? Although the connection maps show some similarities in different animals. It would be good to see to what extent the pattern can be reproduced in the same animal.

Minor concern:
Page 10
Is there any supporting reference for "An eye movement was classified as a saccade if velocity increased beyond 6*std."?

Page 25
What causes the variation of the extant of V1 activation (50-120 mm2)? As a focal brain stimulation method, the spatial specificity of the stimulation is of great concern. So, the possible reason causing the variation of stimulated site should be discussed. Also how can the stimulated site go inside the longitudinal fissure (monkey AL, Figure G, H) is unclear.

Page 25
Figure 1 B shows T2w image (T2 RARE) but no parameter listed in the method part. Please list the parameter in page 6, MRI data acquisition section.

V3d/V3v and V3A have different functionalities. As far as I know, D99 atlas include these 3 areas related to V3 but not a general V3. Please check

Figure 1 E (page 25) and Figure 4 B (page 29). There are large negative responses after opto modulation which is not likely being the post undershoot. Why?

Page 28
Figure 3 A. Boxes on top of spike activity in middle and right panel don't follow presented stimulation paradigm. They should be 10 Hz and 40 Hz but what shows here are 5 Hz.

Please add scale bar for volume space images like Figure 3 D in page 28.

Removing the skull is known to induce signal dropout caused by susceptibility inhomogeneity at the air-brain interface. I encourage authors to show the raw EPI slices to confirm the data quality after surgery and describe how do you consider to solve the issue.

The powers generated by LED were measured by integrating sphere photodiode power sensor. Scattering and absorption loss is always a concern. Because the stimulation was applied epidurally and the targeting depth is layer 4, how effective
power through the dura at the depth of layer 4 is unknown. Other photon related concerns such as photothermal neuromodulation phototoxicity were not well discussed.

Red light experiment is not necessarily a control experiment of heating effect, as also mentioned by authors in page 21. A test on ex-vivo tissue with blue light used in this study might be helpful.

The citation was missing in reference: (Brewer, Press, Logothetis, & Wandell, 2002).
Reference formats are not consistent in the manuscript. Sometimes using numbers but sometimes using author names.

1st Author Response Letter

Response to comments from Editors and Reviewers:

We thank the reviewers for their time and providing us with constructive comments. We believe that addressing these comments for the revised version has greatly improved the overall quality of the manuscript. In what follows we provide a point-by-point response to each comment.

Comments from Reviewer 1:

This paper reports an interesting set of experiments in macaques examining the effect of optogenetic stimulation of V1 in other brain regions (using fMRI) and on behavior (but only in one monkey subject). The authors find that optogenetic stimulation of V1 results in activation of dorsal but not ventral stream extrastriate areas. They relate this to the laminar expression pattern of the opsin, which was predominantly in layer 4B, which projects to MT. Furthermore, they report behavioral data of one monkey showing perceptual detection of the optogenetic stimulation.

This is a well-conducted study, combining optogenetics, histology, electrophysiology, fMRI and behavior. I do not have major technical or methodological concerns but have suggestions to improve the manuscript.

Main comments.

1. It was not clear to me what the monkeys were doing during the fMRI scanning. Were they performing a passive fixation task? Any behavioral control? Behavioral state: were they fully awake? Was there a difference in behavior between the stimulation, i.e. stimulus frequency, conditions? Please clarify.

- fMRI scanning was performed on awake animals; however, there were no task requirements during the stimulation blocks and the animals were free to look around or close their eyes. We and others have recently shown robust visually driven responses under similar free-viewing conditions (eg Ortiz-Rios et al, Neuroimage, 2021; Selvanayaga, J Neurosci, 2019, Russ & Leopold, Neuroimage, 2015). Unfortunately, the eye tracker did not have the sufficient temporal resolution to allow for a precise analysis of eye movement patterns and differences thereof between experimental conditions. We clarify this aspect in the results and methods sections. Thus saccadic eye movements might have been different across the different stimulation conditions. This is a limitation of our study design, which we acknowledge now in the abstract and discussion section.

2. They state that the LED delivered light up to 3.5 mm in depth. It appears that they based this on experiments using agar. However, did they consider that blue light penetrates less deep into the brain?

- We agree with the reviewer that the agar measurements are only an approximation that may not hold for the experimental situation in the living animal. We have therefore removed this sentence from section 3.1 of the manuscript.

3. Although the expression of the opsin is stronger in layer 4B than in other layers, the 40Hz photostimulation produced strong MUA in layer 3. The latter layer project to V2 and then ventral visual

stream areas. Hence, it is not clear why such strong responses in layer 3 did not activate ventral visual stream areas. I think given the discrepancy between the 40Hz electrophysiology data and the histology it is too simplistic to explain the specific dorsal stream activations by the laminar expression pattern of the opsin. This should be discussed in greater detail and in a more nuanced fashion.

- We agree with the reviewer about the gap in our explanation relating the predominant dorsal stream activation, the strongest expression in layer 4B and the more widespread MUA modulation with 40 Hz. Not being able to resolve this discrepancy at this point we can only speculate, which we provide now in section 4.1 of the discussion. In brief, at this point it is possible that there is a greater contribution of neurons that are more sensitive to higher frequency stimulation. Owing to the need for higher temporal resolution to process movement, it is conceivable that such neurons are an exclusive property of the dorsal stream.

4. Figure 4 appears to show activations due to photostimulation in the STS anterior to MT/MST. Are these significant? Can these be shown on coronal sections? Were they present in both banks of the STS?

- All areas shown on the maps are significant with coherence levels > 0.3. We now show coronal slices of the activation clusters in the STS and STG (Figure 4F). Activities are indeed on both banks of the STS and also more anterior to MT/MST.

5. The behavioral data are interesting but somewhat preliminary because they involve only one animal. Furthermore, behavioral effects of V1 optogenetic stimulation in monkeys has been shown before (e.g. recent report by Chen et al., Elife, 2022; which the author should cite and discuss).

- We agree that Chen et al, Elife, 2022 provide an important study and apologize for having omitted it, as our initial manuscript was written before the Chen study was published. We now discuss the Chen study in section 4.3. Briefly, using a masking paradigm Chen et al demonstrate that optogenetic V1 stimulation can influence visual perception, yet it remains unclear whether stimulation by itself produced a phosphene. This is where our study comes in to close this gap, although only at a preliminary level, as indeed only one animal was involved

The authors claim that the monkeys perceive the optogenetic stimulus as a visual stimulus of 5-10% contrast. This is based on the psycho- and chronometric curves of Figure 5C and D. However, the data shown in Figure 5B suggests that even a 5%, and to some extent a 2.5 % contrast visual stimulus produces a difference in behavioral responses to the target compared to the photostimulation. This discrepancy between the two behavioral measures (d' and saccade to target) with respect to the comparison between photostimulation and visual stimulation should be addressed.

- We agree that the results in figure 5B are indicative of a phosphene intensity that resembles a 2.5% contrast visual stimulus. We have updated section 3.6 accordingly. The reason for the difference in estimated contrast across behavioural measures is not clear to us. We are now using more cautionary wording throughout, that optogenetic stimulation most likely resulted in a low contrast phosphene.

Minor comments.

1. The authors show no response to red light stimulation, which they use as a control for heating and other artefacts. However, red light heats neural tissue less than blue light. It would have been better to use as a control stimulation by blue light of a non-injected part of the visual cortex. Nonetheless, it is unlikely that the effects - fMRI and electrophysiology - are due to heating and/or artefacts.

- We agree and include this control suggestion in section 4.2: fMRI measurements under blue light stimulation in a non-transfected cortex would have been a better control for heating (27).‘

2. Eye movement analysis: "We detected eye-movements automatically with a usually employed velocity based algorithm". What is the "usually" employed velocity algorithm?

- We have updated methods section 2.8 with the following:
    - The algorithm we used was based on what is described by Engbert el. 2006.
    - We also added the equations used for the saccade detection threshold.

3. Figure 3: it would be nice to show the exact timing of the stimulation blocks at the top of A.

- Thank you for pointing this out. There was a mistake in the plot and we added the pulse schematic which reflects the timing of the stimulation for each frequency.

Comments from Reviewer 2:

In this paper titled "Optogenetic stimulation of the primary visual cortex selectively drives cortical dorsal stream activity", Ortiz-Rios et al. use optical fibers combined with laminar recording and fMRI in macaque monkeys in order to delineate the functional connectivity of the areas immediately after V1 in the dorsal visual hierarchy. In one monkey, they go beyond and perform a behavioral experiment and demonstrate the effect of optogenetic stimulation of V1 on a psychophysical task, as well as the eye movements and the pupil response.

In general, I find the study carefully designed and meticulously performed. The central question of the paper is of utmost importance in the field. From a technical point of view, the study is impressive given the significant challenges of combining optogenetics with fMRI, neural recordings and behavioral testing in nonhuman primates. Nevertheless, I have some critical concerns, addressing which would increase the quality of the paper.

Major concerns:

1. Red light photons carry ~1.8 electron volts of energy while the blue light photons are in the range 3.1 eV. I am not sure if the energy levels (range of 50mW) reported in the paper are photometric powers measured out of the optical fiber or electrical power levels used to drive the LEDs? In other words, I don't know if the red (control) vs blue (test) powers are equated for the total photo power that was injected into the tissue or for the electrical power that was used to drive the LEDs? If the former, then the electrical power must have been higher for the red LEDs to compensate for the weaker photons (when heating is to be controlled), is that true?

- The power was measured at the fibre tip before being coupled to the probe for intracortical stimulation and before being placed on top of the tissue for surface stimulation. All power levels were measured with a photodiode power sensor and a digital power meter (Thorlabs PM100D). The power level controls on the laser system were adjusted till the desired power, measured by the photodiode, is achieved. The optical stimulation section 2.3 of the manuscript was adjusted accordingly.

2. On a very related and perhaps more important point, long wavelength light is absorbed in the body tissue and blood far less than the short wavelengths. This can be easily demonstrated by covering the tip of red and green laser pointers with one's thumb; the green doesn't go through but the red illuminates the entire thumb. This in fact is the reason for developing red-shifted opsins in order to allow further light penetration in the tissue. Further penetration means less absorption and less heating, this jeopardizes the red light as a control for heating, which is a serious concern here given the long durations of optical stimulation. The authors are probably aware of this issue as they have mentioned in the paper that the ideal control experiment here is using the blue light on intact neural tissue (not transduced). I don't have the numbers, but if the absorption of the red light in a given volume of tissue around the fiber tip is less than the blue light by less than 1 order of magnitude, it could serve as a partial control as it would be possible to estimate the upper bound of a possible heating effect. But I doubt if that is true. Either way, I believe this shortcoming should be mentioned and addressed in the paper. I agree with the analysis of the authors that the sensitivity of the observed effect to temporal frequencies at relatively high rates cannot be explained with heating given its sluggish temporal response. I think this point should be inflated, the point about the red being a control, deflated.

- We thank the reviewer for this suggestion. We have performed ex vivo tissue heating experiments with red and blue light and reported the results in our reply to reviewer 3.

3. The animals were first trained with a visual stimulus, then they entered a 3 conditioned task that included 33.3% brain stimulation trials. It is possible to argue that the animals did not perceive the optogenetic stimulation, but their brain circuit rewired and they learned a new rule to perform this new class of trials bypassing visual perception. Showing the performance of the first day when cortical stimulation is introduced can be helpful in this regard. Did the monkey perform the task at chance for the optogenetic trials in the first day then gradually learned it over the course of multiple days? Or was there above chance performance even in the first day? That would imply easy generalization of the visual task to the optogenetic task and help the discussion specially in the absence of strong eye movement evidence in support of the phosphene theory.

Thank you for this suggestion. Below and as supplementary figure 13A, we provide information on d' from the very first optogenetics session. Thus the monkey generalized from the visual to the optogenetic task from the first day on.

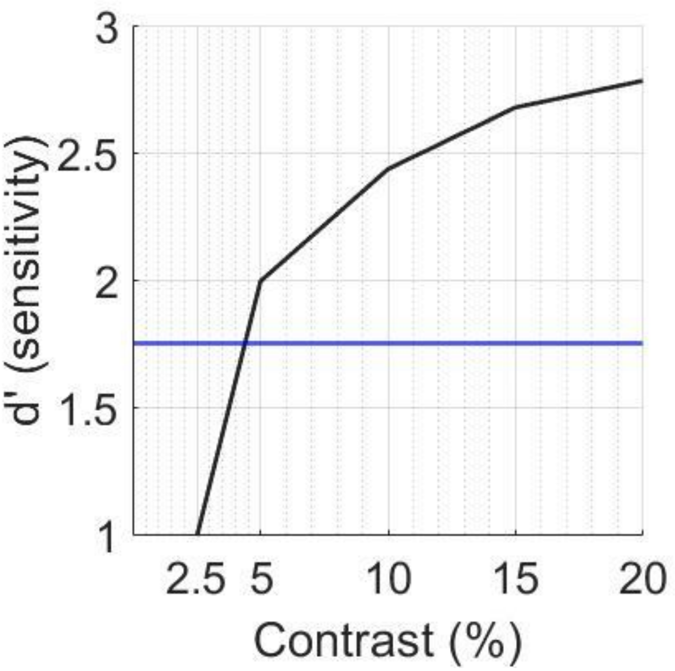

4. The design feature that allows free eye movements before landing at a response targets is brilliant. Its results though need to be more clearly communicated. Specifically, it is not clear what happens to the saccadic eye movements when the optogenetic stimulation is applied. The authors show that saccades to the RF are less common than the high contrast stimulus and not different from the low contrast stimulus, but are they different from the no stimulus condition? Figure 5 A shows a trace of an effect, but I failed to find a quantitative analysis of that small effect. The question is basically: is there any significant bias for saccading to the RF? Also see my minor comment #2. Related comment: my hat off to the amazing pupil effect.

- We respond to this comment further below under minor comment 2.

Minor concerns:

1. Technically, the behavioral task used here is not a 2AFC. Apart from having 2 alternatives, what is referred to as the two alternative forced choice design is a specific design (not necessarily good for this study) that aims at zeroing the effect of choice biases. In a 2AFC, both stimulus conditions are presented in each trial (side by side or in temporal sequence) and the subject is asked to choose which one (left or right, first or second, etc.) contained a given signal (eg brain stimulation). In this way, even if the animal constantly chooses one response target, it will receive only 50% reward. The design used here is not 2AFC as it only has one stimulus in each trial and its chance level vary based on choice bias. Note that if the monkey chooses to pick the left target all the time he will receive 66% reward, but if he picks the right target all the time he will receive only 33%. It is OK as d' is used here for measuring performance, but the task is not 2AFC.

- Indeed this task is fundamentally a Yes/No task with added components to differentiate visual detection from any potentially induced oculomotor response. Following the reviewer's recommendation we have now renamed the task throughout the text.

- The reviewer is correct that in principle a bias could have been introduced in the way the task was presented. However this was not the case in monkey FL. From the first day of optogenetic testing, she generalised performance from visual to optogenetic trials (Figure 5A, supplementary figure 13A) with approx. 80% correct performance for high visual contrast, optogenetics and catch trials. Across experiments, the tendency to go to the left 'yes' side was approx. 20% during catch trials (supplementary figure 13B) was well below the approx. 60% performance during optogenetics. This explains the overall good d' estimate that we obtained for the study (figure 5C).

2. Subplot 5.b shows the ratio of direct saccades to the target, but it would be more informative to plot the saccades to the target/RF.

- Thank you for this suggestion. In supplementary Figure 13C, we show the proportion of saccades to the RF before executing a 2nd saccade to the response target. In these false alarm trials, the monkey almost always executed a direct saccade to the response target and thus avoided looking at the RF. Importantly, this pattern occurs only in approx. 20% of all catch trials (supplementary Figure 13B)

3. In the discussion, in the review of optogenetic studies regarding V1 and phosphenes Rajalingham et al., 2021, Nature Methods is missing. There, using an optical array we have shown that optogenetic silencing in V1 induces transient scotomas in the corresponding part of the visual field in the form of zones of elevated threshold in a direct contrast comparison task between two targets. The results are very related to this study and I am curious to see how the effect sizes reported here compare to the opposite direction effect sizes from silencing.

- Thank you for pointing out this study. It wasn't published yet when we first wrote our manuscript and we apologise for not having it included in the meantime. Indeed, the findings by Rajalingham et al are highly relevant and the effect sizes seem quite similar. We have added the paper to our discussion section.

4. In the introduction the primate brains are referred to as "more evolved" in comparison to rodent brains. Well, throughout the human history we have enjoyed putting ourselves on top of an evolutionary ladder and I don't mind the authors sitting there, but I humbly bring it to attention that evolution doesn't have a direction, it is an ever changing network and our street rats are as "evolved" (whatever that term means) as us, but in other dimensions. I would prefer a more objective term like: large primate brains, larger and more complex primate brains, etc.

- We agree with the reviewer's thinking and have adopted the text accordingly towards primate brains.

Comments from Reviewer 3:

This study describes an exciting application of the cutting-edge optogenetic-fMRI technology to reveal brain wide cell-specific connections in non-human primates (NHP). Although application of optogenetic stimulation mapping brain functional networks has been conducted in several animal model, applying optogenetic methods to primate brain is challenging. Thus far, only a few studies successfully to manipulate behavior in NHP using optogenetics. Here, the authors demonstrated a large-scale circuit

connection induced by optogenetic stimulation using fMRI. To confirm the effectiveness of the stimulation, multiple methods including electrophysiology, immunohistochemistry and behavior were applied. The succeed in achieving optogenetic manipulation and network mapping in macaque provide referential value for the neuroscience community and highly benefit translational neuroscience.

Details of the study were comprehensively described and statistical analyses were properly performed. I only have three major concerns and some minor comments as detailed below.

Major concern:

1. The definition of connected site location is suspected. Although the activation location of MT in volume view is convincing (Fig. 3 D), the activations on surface view seems have a mismatch (Fig. 4 E). The MT/MST is located in the superior temporal sulcus. However, there is an obvious connected site located in the gyrus between lunate sulcus (LS) and superior temporal sulcus (STS) where seems to be V4. This is also true in Supp. Fig. 10 and graphical abstract.

In addition, there are also other areas been activated beside V2, V3 and MT/MST. For example, in figure 4 E, there are highly activated clusters in the Postcentral gyrus (maybe somatosensory cortex) and anterior end of temporal cortex (maybe TEa). The authors only describe these areas in dorsal stream but neglect of other areas which is too subjective.

- We have now updated figure 4 to illustrate the activation cluster in coronal slices. These now include the clusters found in areas STS, STG, V4, anterior area TAa and somatosensory area 3a/b among others. The corresponding slices are accompanied by the respective atlas parcellation from D99.

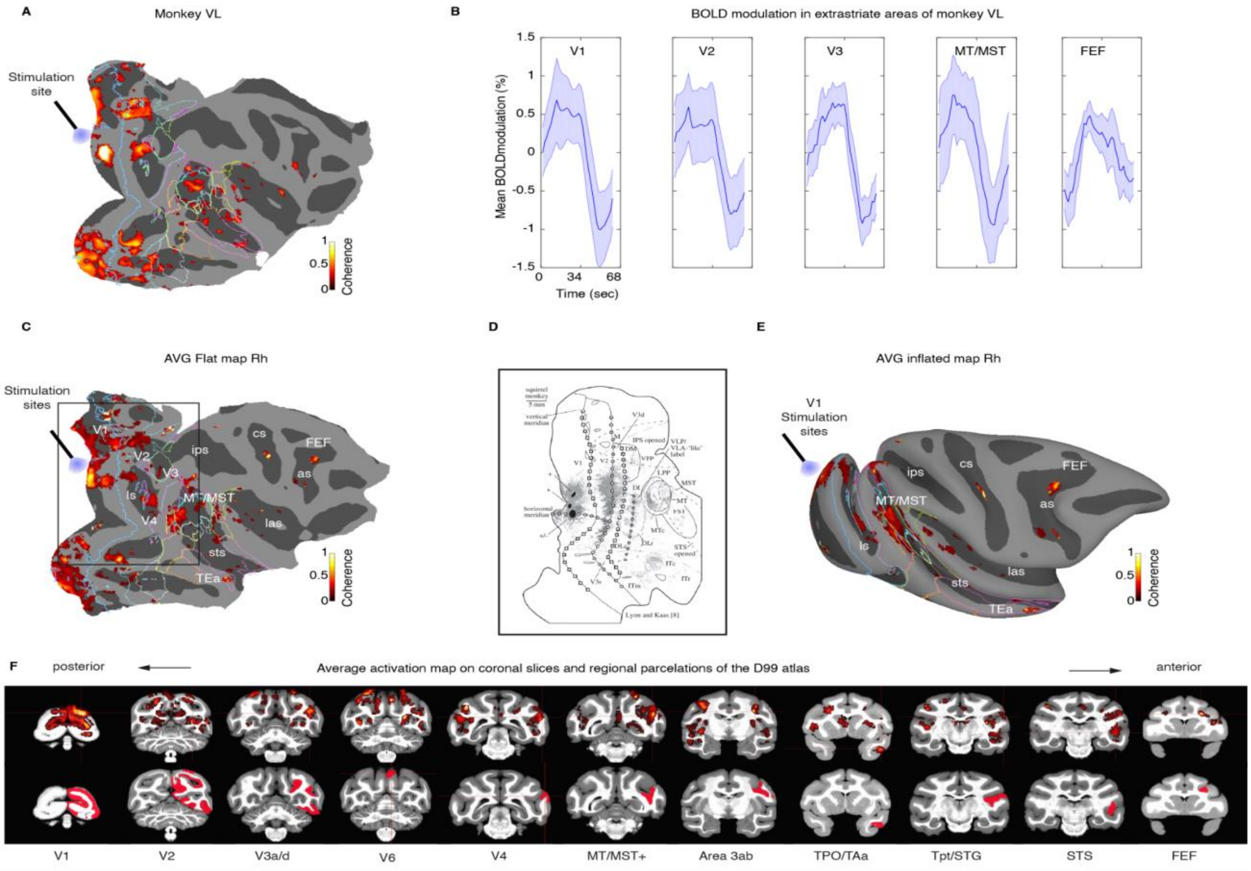

Moreover, V2 has different compartments selective to either ventral stream or dorsal stream such as "color" stripes and "motion" stripes. The activation map here can't define which compartment they are.

With saying this, the hypothesis that "optogenetics induced projection along the cortical dorsal stream" is also questioned, because V1 layer 4 also been reported to selective to orientation. Orientation domains were also detected in ventral stream areas like V4.

In general, from both the results and hypothesis side, the conclusion of "selectively drives cortical dorsal stream activity" looks overstated.

- We agree with the reviewer about the different compartments of V2, which unfortunately are beyond our present fMRI resolution. We, therefore, adopt more cautionary wording throughout the manuscript, beginning with the title and abstract.

For better understanding of these maps, the name of the important sulcus (e.g., LS and STS) should be labeled as a marker of geometry. Please also indicate the color indices of gyrus and sulcus. I assume the surface follows Freesurfer convention that dark gray is sulcus and light gray is gyrus. It would also be helpful to only keep the D99 atlas borders that related to the activations in this study. The current figure, with so many areas, is not easy for readers to localize the activations.

- We thank the reviewer for the suggestion. We have now updated figure 4 to clarify sulci and gyri and followed the classical Freesurfer light/dark grey colour scheme. Additionally, we also plot contours of only visual areas in the 3D and flat maps to clarify areal activations.

2. The rational to use the coherence analysis mentioned by authors is "the hemodynamic response function (HRF) of opto-fMRI in macaques has not been formally characterized". This rational doesn't sound strong to me because the block design used in the fMRI study should be less influenced by HRF. Although the coherence analysis has been used in many studies, the GLM analysis method is still the most conventional method to detect activations. As probably been one of the first few papers to show optogenetic induce brain connections in MRI, I suggest author also show the activation map from conventional GLM analysis, at least in supplemental. From methodological view this will be help to boost further opto-fMRI studies and the understanding of optogenetic BOLD physiology.

- We thank the reviewer and agree that the hemodynamic responses for opto-driven responses might be less influenced by the block design and have now added the results of the GLM into the supplementary for comparison. However, we opted for caution given the unknown HRF parameters that might arise from optogenetic stimulation and instead preferred to use a model-free method as done by the first Opto fMRI paper.

Lee JH, Durand R, Gradinaru V, Zhang F, Goshen I, Kim DS, Fenno LE, Ramakrishnan C, Deisseroth K. Global and local fMRI signals driven by neurons defined optogenetically by type and wiring. Nature. 2010 Jun 10;465(7299):788-92. doi: 10.1038/nature09108. PMID: 20473285; PMCID: PMC3177305.

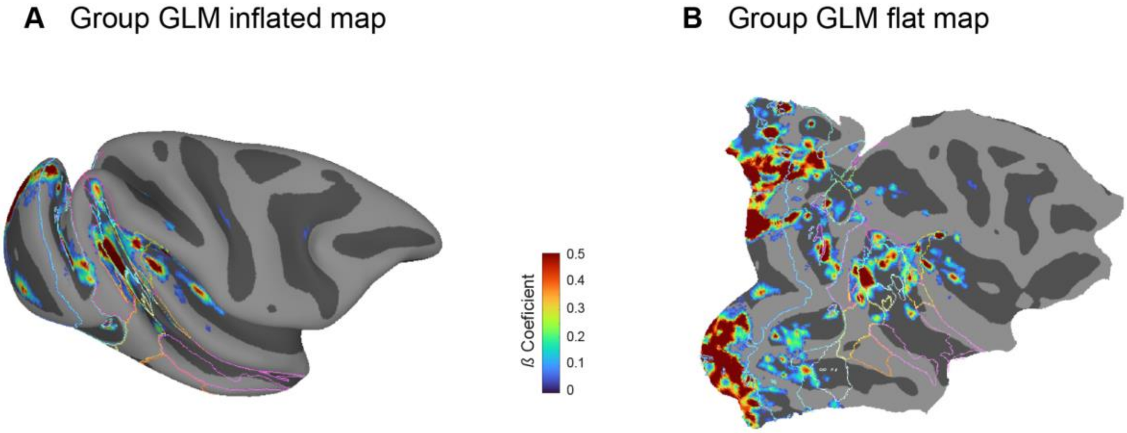

.

Supp.Fig.12 General linear model average activation map.

As the input dataset to 3dDeconvolve, we provided the detrended time series and then applied ordinary least squares to a regression model of the BOLD signal which included a gamma variate block design of the hemodynamic response to the opto stimulation periods (30 secs on and off). A general linear T-tests were performed between opto stimulation and the no-stimulation periods. The stats output from 3dDeconvolve was then used to visualize the activation areas. The threshold was chosen to be at a significant p-value < 0.01. We confirmed significant activation (T-value colourmap range 1.9 < 8.3) across visual and dorsal stream-related regions in the average map. Maps are displayed on the inflated surface (A) and the flat map surface (B).

3. What's the level of reproducibility of these large-scale cortical circuits induced by optogenetic stimulation? Although the connection maps show some similarities in different animals. It would be good to see to what extent the pattern can be reproduced in the same animal.

- We were able to observe the same V1 activation pattern on the same subject (see example below of 2 different sessions of the same reproduce animal).

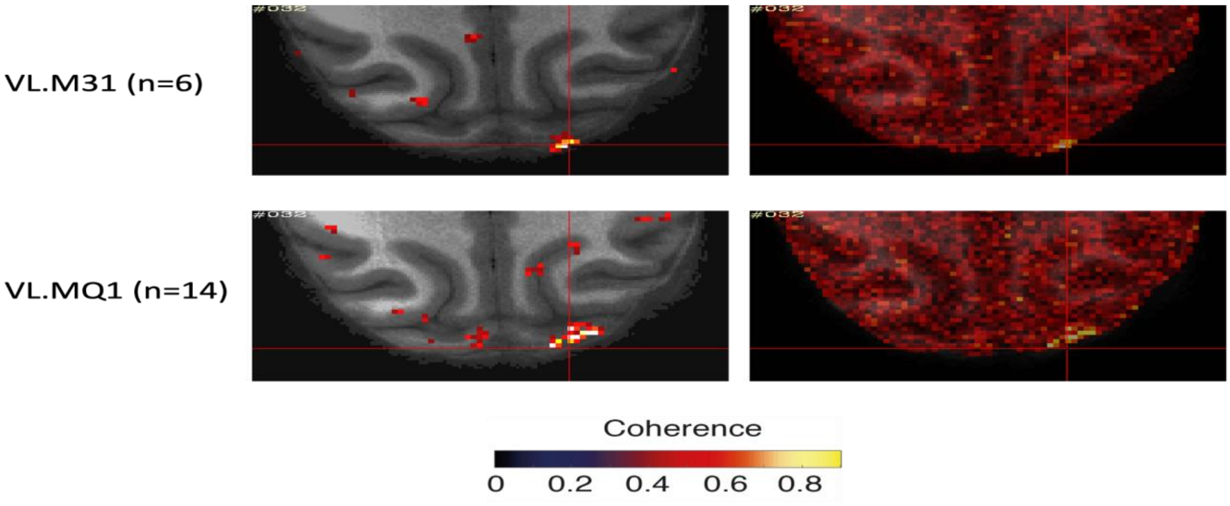

Minor concern:

Page 10

Is there any supporting reference for "An eye movement was classified as a saccade if velocity increased beyond 6*std."?

- Thanks for pointing this out. The saccade detection algorithm was based on the work of Engbert el. 2006. We have also added the used equation in the methods section.

Page 25

What causes the variation of the extant of V1 activation (50-120 mm2)? As a focal brain stimulation method, the spatial specificity of the stimulation is of great concern. So, the possible reason causing the variation of stimulated site should be discussed. Also how can the stimulated site go inside the longitudinal fissure (monkey AL, Figure G, H) is unclear.

- In the manuscript discussion (section 3.5) we now state: "At the cortical level in V1, we observed some variation across animals in the overall cluster extent (50 - 120mm. This could be due to several factors, such as the transfection spread of AVV9 across the cortical sheet and or the polysynaptic propagation of functional activity."

Page 25

Figure 1 B shows T2w image (T2 RARE) but no parameter listed in the method part. Please list the parameter in page 6, MRI data acquisition section.

- We have now updated the manuscript to include these additional acquisition parameters.

V3d/V3v and V3A have different functionalities. As far as I know, D99 atlas include these 3 areas related to V3 but not a general V3. Please check

- Activation in V3 was observed in both V3d and V3a. We have now relabelled the activation of area V3 to clarify specific nomenclature. See figure 4F.

Figure 1 E (page 25) and Figure 4 B (page 29). There are large negative responses after opto modulation which is not likely being the post undershoot. Why?

- Indeed, this is not an undershoot of the BOLD signal, this is just modulation around the mean. Different software packages implement different scaling processes. In AFNI scaling is performed voxel-wise to keep signal interpretable as modulation % signal change around the mean of each time-series. Similar modulation for visual responses could be observed in Supplementary Figure 3. For more specific details see (Chen G, Taylor PA, Cox RW (2017). Is the statistic value all we should care about in neuroimaging? Neuroimage 147:952-959).

Page 28

Figure 3 A. Boxes on top of spike activity in middle and right panel don't follow presented stimulation paradigm. They should be 10 Hz and 40 Hz but what shows here are 5 Hz.

- Thanks for pointing out this typo. The stimulation pulses schematic was fixed for 10Hz and 40Hz in figure 3.

Please add scale bar for volume space images like Figure 3 D in page 28.

- Figure 3D has been updated with a scale bar.

Removing the skull is known to induce signal dropout caused by susceptibility inhomogeneity at the air-brain interface. I encourage authors to show the raw EPI slices to confirm the data quality after surgery and describe how do you consider to solve the issue.

- We are well aware of this issue. However, we solved this issue prior to our imaging experiments. For the reviewer's interest, we show below an example of raw EPI images of one animal with the crosshair at the site of the stimulation/ chamber region. We solve distortion induced by the changes of high signal (e.g. brain) to air gap (e.g. chamber) by filling the chamber with silicone material. The small distortion we observed in the left side of the brain near V1 is due to a ceramic screw. In one animal only we observed an unresolved distortion in the area of implantation.

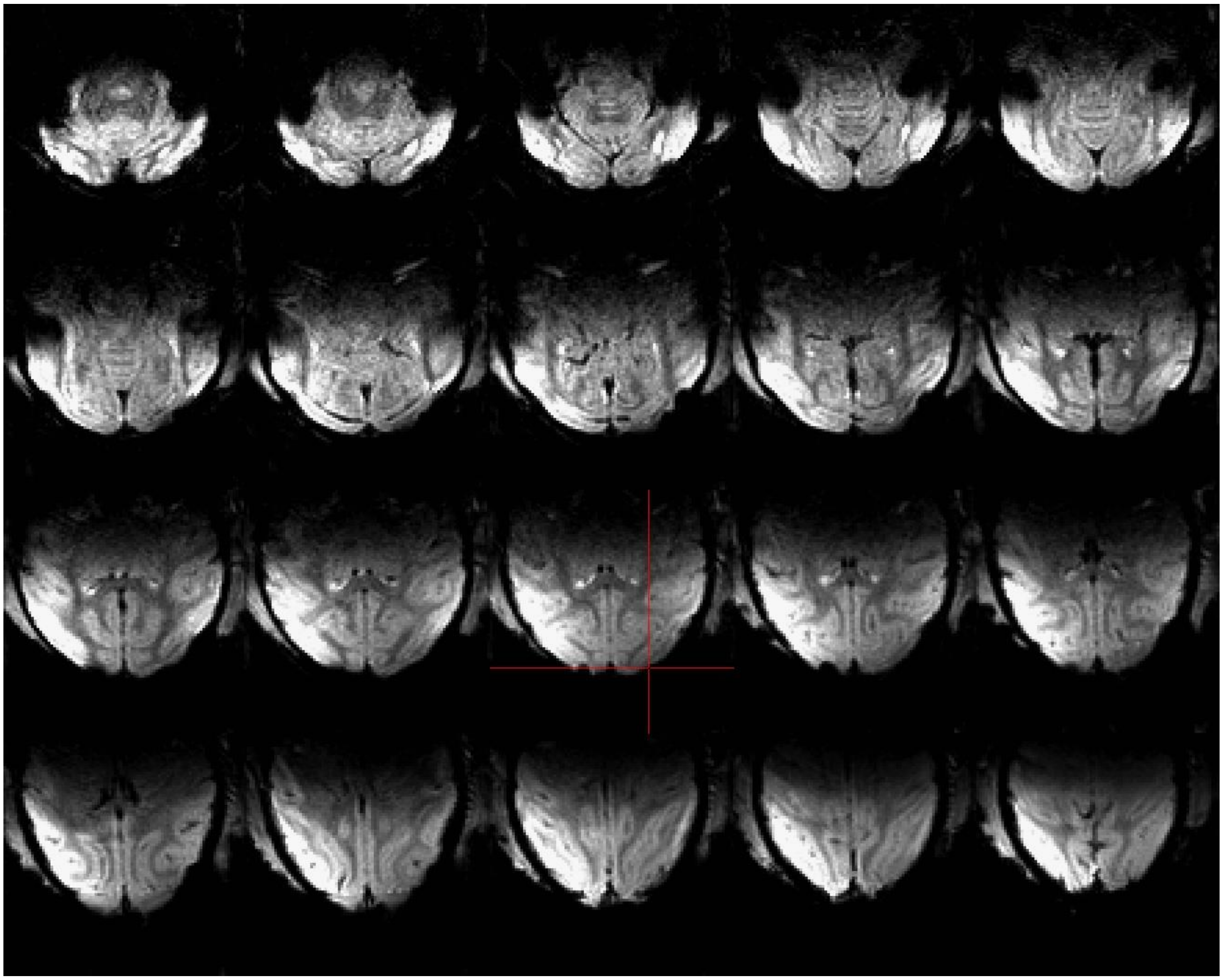

The powers generated by LED were measured by integrating sphere photodiode power sensor. Scattering and absorption loss is always a concern. Because the stimulation was applied epidurally and the targeting depth is layer 4, how effective power through the dura at the depth of layer 4 is unknown. Other photon related concerns such as photothermal neuromodulation phototoxicity were not well discussed.

- We could not examine the phototoxicity within the scope of this paper. However, we would expect the small fraction of the light reaching the tissue (due to light absorption and the halving of light intensity every 200um (Aravanis et al. 2007)) not to be harmful. During histological analysis, we did not observe any signs of damage other than due to electrode insertion.

- As for light reaching layer 4, the low latency of the observed optogenetic modulation confirms that light reaches the deeper layers. However, we were not able to measure the exact amount of light reaching the deeper layers.

- We do not believe that the observed modulation is due to thermal effects. To begin, the modulation latencies are low and within what is expected from ChR2 while thermal neural modulation latencies are much longer (Horváth et al., 2020; Owen et al., 2019). Additionally, we performed an ex-vivo experiment to measure the temperature increases due to both red and blue LED in neural tissue ex-vivo; we found that temperature increases are minor few millimetres away from the optical fibre and that the temperature increases are not wavelength specific (Supp. Fig. 11) while our neural modulation is wavelength specific. Therefore, we can say with a high degree of certainty that the observed neural modulation is due to optogenetic modulation via ChR2 activation and not due to thermal effects. We have added the supplementary figure and adjusted the text in the discussion accordingly in section 4.2.

Red light experiment is not necessarily a control experiment of heating effect, as also mentioned by authors in page 21. A test on ex-vivo tissue with blue light used in this study might be helpful.

- We thank the reviewer for raising this important point about temperature effects. There is indeed a rich literature on this topic (eg Lee, Nature 2010; Desai, J Neurophys, 2011; Christie et al, Neuroimage, 2013; Stujenske et al, Cell Reports, 2015; Arias-Gil, Neurophotonics, 2016; Schmid et al, MRM, 2017; Owen et al., Nat Neuro, 2019). In a nutshell, light can heat up non-transfected neural tissue and result in **decreased** spiking and **negative** BOLD activity under the fiber tip. In contrast, we observed < 10ms latency **increased** spiking and **positive** BOLD activity under the fiber tip.

Some of the most important temperature modulating variables are the size of the fiber and stimulation parameters such as wavelength, amplitude, frequency and duty cycle. While unfortunately we are at present not able to carry out a control experiment that applies our stimulation approach to non-transfected tissue, we have performed ex-vivo temperature measurements (details are provided in supplementary figure 11). As expected, we found indeed a temperature modulation that closely follows the 30s on/off stimulation sequence. The amplitude of this modulation was approx. 0.7°C at 1 mm and approx. 0.1°C at 5 mm distance for pulsed stimulation. Importantly, we did not measure any temperature difference for 5 vs 40 Hz and 451 vs 626 nm. This contrasts with our fMRI finding of BOLD modulation that is stronger for 40 compared to 5 Hz and stronger for 451 compared to 626 nm.

From these considerations we believe that temperature modulations are unlikely the major driving effect of our fMRI results. At the same time, we cannot entirely rule out temperature contributions. We have therefore included details about temperature aspects throughout the revised text and use more cautionary wording.

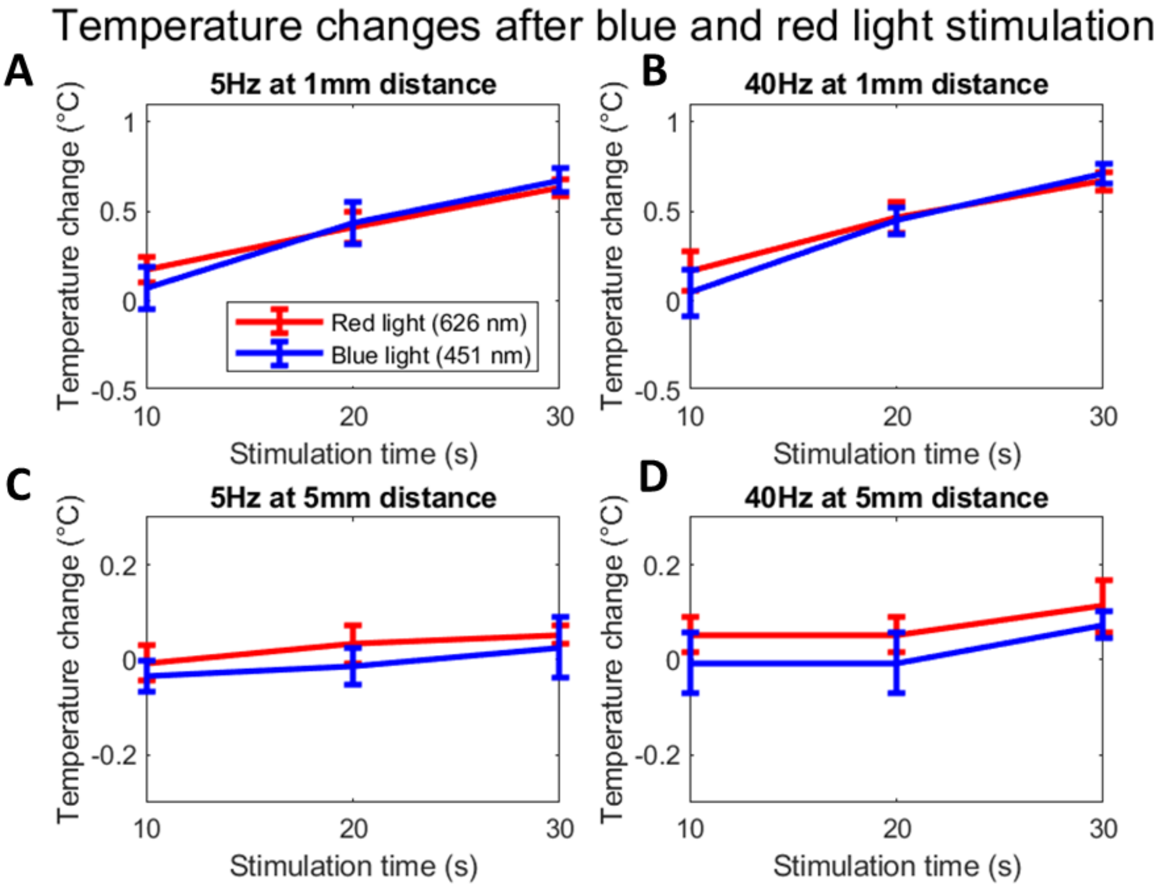

Supplementary Figure 3. Mean temperature changes of ex vivo neuronal tissue in response to stimulation with blue and red light at 50mW over 30 seconds (5 repetitions). A temperature probe was inserted into the neuronal tissue at two different distances from the surface of the electrode (1mm and 5mm). Temperatures were measured using a digital thermometer (Fluke 51). Temperature changes were calculated as differences from the baseline period during which there was no stimulation. A. Temperature changes measured inside the tissue at 1 mm distance from the LED after stimulation with blue and red light pulsed at 5Hz. B. Same as A but with a frequency of 40Hz. C. Temperature changes measured inside the tissue at 5 mm distance from the LED after stimulation with blue and red light at 5Hz. D. Same as C but with a frequency of 40Hz.. Note the y-axis scale difference between panels A & B vs C & D.

The citation was missing in reference: (Brewer, Press, Logothetis, & Wandell, 2002).

Reference formats are not consistent in the manuscript. Sometimes using numbers but sometimes using author names.

- Thank you for pointing this out. The reference was added and the rest of the in-text citations were fixed to be uniform.

Accept Letter

Dear Prof Schmid,

Thank you for submitting your manuscript to Current Research in Neurobiology. I am pleased to inform you that your manuscript has been accepted for publication. My comments, and any reviewer comments, are below.

Your accepted manuscript will now be transferred to our production department. We will create a proof which you will be asked to check, and you will also be asked to complete a number of online forms required for publication. If we need additional information from you during the production process, we will contact you directly.

We appreciate and value your contribution to Current Research in Neurobiology. We regularly invite authors of recently published manuscript to participate in the peer review process. If you were not already part of the journal's reviewer pool, you have now been added to it. We look forward to your continued participation in our journal, and we hope you will consider us again for future submissions.

CRNEUR aims to be a unique, community-led journal, as highlighted in the Editorial Introduction. As part of this vision, we will be regularly seeking input from the scientific community and encourage you and your co-authors to take the survey.

We would also like to invite you to take part in our CRNEUR Author Question & Answer (Q&A), which could get published alongside your article and help to promote it. We suspect you might have an interesting story of perseverance or team work that was required for the research study to complete, or a diversity of perspectives that you might share, as a way of inspiring others about neuroscience.

Kind regards,

Yogita Chudasama
Associate Editor
Current Research in Neurobiology

Editor and Reviewer comments:

Reviewer 1: The authors have addressed all my comments and I have no further concerns.
Reviewer 2: The authors have successfully addressed all of my comments. I have no further comments and find the paper ready for publication.

-------- *End of Review Comments* --------
